# Supplementary material for: Stimuli-responsive rotaxane-branched dendronized polymers with tunable thermal and rheological properties
Source: Nat Commun. 2023 Aug 31;14:5307. doi: 10.1038/s41467-023-41134-8 (PMC10471591; doi:10.1038/s41467-023-41134-8)
Supplement: Supplementary file 1 — Supplementary Information [file 41467_2023_41134_MOESM1_ESM.pdf]

Supplementary Information

Stimuli-responsive Rotaxane-branched Dendronized Polymers with Tunable Thermal and Rheological Properties

Yu Zhu[1], Hanqiu Jiang[2,3], Weiwei Wu[4], Xiao-Qin Xu[1], Xu-Qing Wang[1,*], Wei-Jian Li[1], Wei-Tao Xu[1], GengXin Liu[4], Yubin Ke[2,3], Wei Wang[1,*] & Hai-Bo Yang[1*]

[1] Shanghai Key Laboratory of Green Chemistry and Chemical Processes, School of Chemistry and Molecular Engineering, East China Normal University, Shanghai 200062, P. R. China

[2] Spallation Neutron Source Science Center, Dongguan 523803, China

[3] Institute of High Energy Physics, Chinese Academy of Sciences (CAS), Beijing 100049, China

[4] State Key Laboratory for Modification of Chemical Fibers and Polymer Materials, Center for Advanced Low-dimension Materials, College of Material Science and Engineering, Donghua University, Shanghai 201620, China

* Corresponding authors.

E-mail: xqwang@chem.ecnu.edu.cn (X.-Q. W.); wwang@chem.ecnu.edu.cn (W. W.); hbyang@chem.ecnu.edu.cn (H.-B. Y.)

Table of Contents (48 Pages)

1. Supplementary materials and methods……………………………..………………….………......S3

2. Synthesis and characterizations of macromonomers MGn…………..……………………….S5

3. Anion-induced thickness modulation of macromonomers MGn……..………….……….….. S28

4. Synthesis and characterizations of rotaxane-branched DPs………………………………… S32

5. Small angle neutron scattering (SANS) experiments…...…….…S38

6. AFM images of rotaxane-branched DPs …………………………………………………... S41

7. Anion-induced thickness modulation of rotaxane-branched DPs..……………………..….…S43

8. Tunable thermal properties of rotaxane-branched DPs…………….……. ……...………S45

9. Tunable rheological properties of rotaxane-branched DPs....................…….…….….……S47

10. Supplementary references………………………………………………………….......S48

1. Supplementary materials and methods

All reagents were commercially available and used as supplied without further purification, compounds **S1-S9,** [2]rotaxane **1** and Grubbs' 3rd generation catalyst (**Ru-III**) were prepared according to the published procedures[1-3]. For these reported compounds **S1-S9**, only ^{1}H NMR spectra were measured and compared with those in literatures to confirm their structures. Deuterated solvents were purchased from Cambridge Isotope Laboratory (Andover, MA).

All solvents were dried according to standard procedures and all of them were degassed under N_2 for 30 minutes before use. All air-sensitive reactions were carried out under inert N_2 atmosphere. ^{1}H NMR, ^{13}C NMR and ^{31}P NMR spectra were recorded on Bruker 300 MHz Spectrometer (^{1}H: 300 MHz, ^{31}P: 122 MHz; ^{13}C: 75 MHz), Bruker 400 MHz Spectrometer (^{1}H: 400 MHz; ^{31}P: 162 MHz; ^{13}C: 101 MHz), Bruker 500 MHz Spectrometer (^{1}H: 500 MHz; ^{31}P: 202 MHz; ^{13}C: 126 MHz) at 298 K. The ^{1}H and ^{13}C NMR chemical shifts are reported relative to residual solvent signals, and ^{31}P {^{1}H} NMR chemical shifts are referenced to an external unlocked sample of 85% H_3PO_4 (δ 0.0). The MALDI MS experiments were carried out on a Bruker UltrafleXtreme MALDI TOF/TOF Mass Spectrometer (Bruker Daltonics, Billerica, MA), equipped with smartbeam-II laser. Electrospray ionization (ESI) mass spectra were recorded with a Waters Synapt G2 mass spectrometer. Gel permeation chromatography (GPC) was carried out at 40 °C using THF as the eluent with a flow rate of 1.0 mL min^{-1}, and the system was calibrated with polystyrene standard. The absolute molecular weight of polymers were determined using high-performance size-exclusion chromatography (HPSEC), Viscotek (Viscotek TDAmax) with a differential viscometer (DV), right angle laser-light scattering (RALLS, Viscotek), low-angle laser-light scattering (LALLS, Viscotek), and refractive index (RI) detectors. The column set consisted of a PL 10 mm guard column (50 × 7.5 mm^2) followed by one Viscotek T6000 column (8.0 × 300 mm, 10 mm bead size; 104 Å pore size) and one Viscotek T4000 column (8.0 × 300 mm, 6 mm bead size; 1.5 × 103 Å pore size). Differential scanning calorimeter (DSC) was performed on a Q2000 DSC system in nitrogen atmosphere. An indium standard was used for temperature and enthalpy calibrations. All the samples were first heated from -40 to 140 °C and held at this temperature for 3 min to eliminate the thermal history, and then, they were cooled to -40 °C and heat again from -40 to 140 °C at a heating or cooling rate of 10 °C min^{-1}. All the AFM images were obtained on a Dimension Fast Scan (Bruker), using ScanAsyst mode under ambient condition,

the samples were prepared by spin casting dilute solutions (10^{-4} mg mL^{-1}) in THF onto freshly cleaved mica for the polymers.

2. Synthesis and characterizations of macromonomers MGn

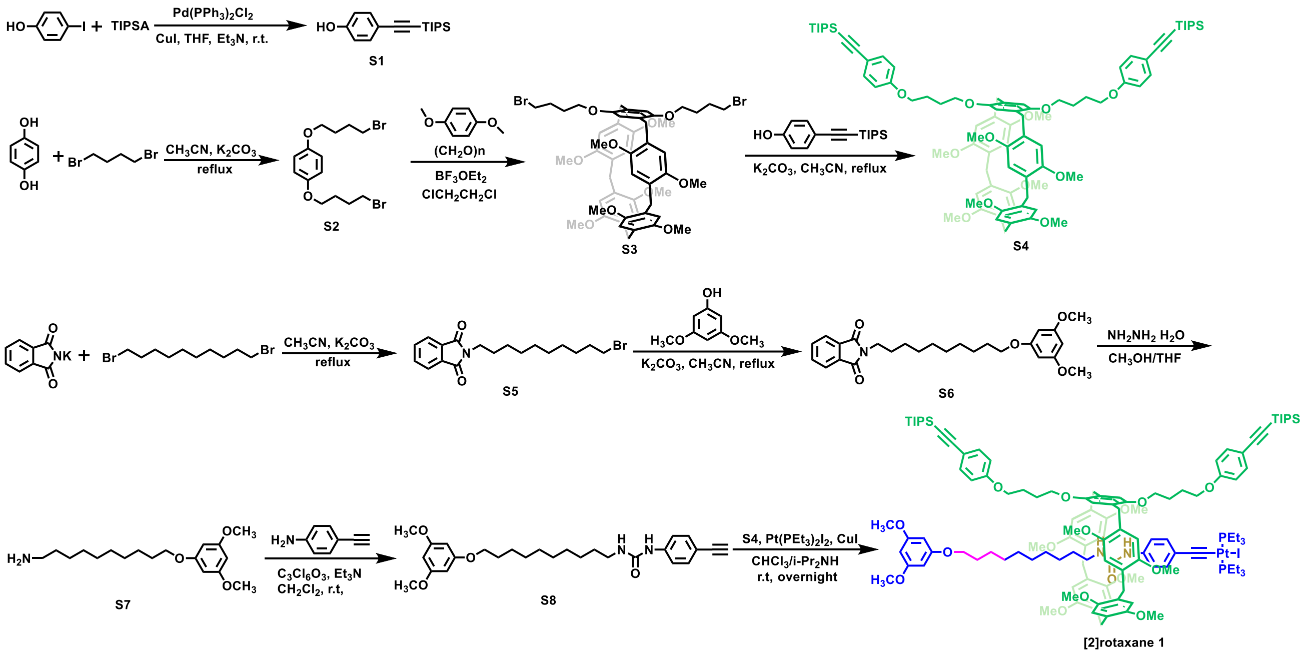

Supplementary Fig. 1 The synthesis route of [2]rotaxane **1**.

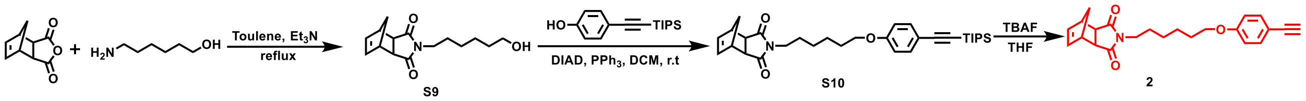

Supplementary Fig. 2 The synthesis route of **2**.

Synthesis of S1: A mixture of 4-iodophenol (5.0 g, 22.7 mmol), triisopropylsilylacetylene (10.2 mL, 45.4 mmol), $Pd(PPh_3)_2Cl_2$ (1.5 g, 2.2 mmol) and CuI (0.43 g, 2.2 mmol) were added in a Schlenk flask, the Schlenk flask was then evacuated and back-filled with N_2 three times. Next, dry THF/Et_3N(v/v, 30/60 mL) was added. The reaction was stirred for 12 hours at room temperature. the reaction mixture was filtered and the filtrate was concentrated in vacuum. The resultant residue was purified by column chromatography with DCM-PE (1:1 in volumetric ratio) as eluent to afford compound **S1** as a pale-yellow solid (4.6 g, 74% yield). ^{1}H NMR (400 MHz, $CDCl_3$, 298 K): δ 7.37 (d, J = 8.6 Hz, 2H), 6.75 (d, J = 8.6 Hz, 2H), 5.17 (s, 1H), 1.12 (s, 21H).

Synthesis of S2: Mixing hydroquinone (5.5 g, 50 mmol) and 1,4-dibromobutane (19.7 mL, 300 mmol) in acetonitrile, then K_2CO_3 (30.1 g, 200 mmol) was added into the reaction flask. The resultant suspension was refluxed at 88 °C overnight. After cooling to room temperature, the reaction mixture was filtered and the filtrate was concentrated in vacuum. The resultant residue was purified by column chromatography with DCM-PE (1:6 in volumetric ratio) as eluent to afford compound **S2** as a white

solid (7.6 g, 40% yield). ^{1}H NMR (400 MHz, $CDCl_3$, 298 K): δ 6.81 (s, 4H), 3.94 (t, J = 6.1 Hz, 4H), 3.48 (t, J = 6.6 Hz, 4H), 2.10-2.03 (m, 4H), 1.96-1.87 (m, 4H).

Synthesis of S3: Under N_2 atmosphere, mixing **S2** (1.5 g, 4.0 mmol), 1,4-methoxybenzene (4.9 g, 36 mmol) and paraformaldehyde (1.19 g, 40 mmol) in 1,2-dichoroethane (100 mL), then boron trifluoride diethyl etherate (5.0 mL, 40 mmol) was added to the solution. The reaction was stirred for 16 hours at room temperature, the reaction mixture was concentrated in vacuum. The resultant residue was dissolved in CH_2Cl_2 and washed with water, the organic layer was dried over anhydrous Na_2SO_4 and evaporated to afford the crude product, which was isolated purified by column chromatography with DCM-PE (1:1 in volumetric ratio) as eluent to afford compound **S3** as a white solid (1.2 g, 30% yield). ^{1}H NMR (300 MHz, $CDCl_3$, 298 K): δ 6.82-6.76 (m, 10H), 3.95-3.70 (m, 38H), 3.69-3.62 (m, 4H) 1.76-1.60 (m, 8H).

Synthesis of S4: Mixing compound **S3** (2.98 g, 3 mmol) and **S1** (3.36 g, 12 mmol) in acetonitrile (120 mL), then K_2CO_3 (3.30 g, 24 mmol) was added into the reaction flask. The resultant suspension was refluxed at 88 °C overnight. After cooling to room temperature, the reaction mixture was filtered and the filtrate was concentrated in vacuum. The resultant residue was purified by column chromatography with DCM-PE (1:5 in volumetric ratio) as eluent to afford compound **S4** as a white solid (2.5 g, 60% yield). ^{1}H NMR (400 MHz, $CDCl_3$, 298 K): δ 7.44-7.39 (d, J = 12.1 Hz, 4H), 6.84-6.76 (m, 14H), 4.01-3.63 (m, 42H), 1.98- 1.95 (m, 8H), 1.14 (s, 42H).

Synthesis of S5: Mixing phthalimide potassium salt (9.25 g, 50 mmol) and 1,10-dibromodecane (22.5 g, 75 mmol) in acetonitrile (300 mL), then K_2CO_3 (8.3 g, 60 mmol) was added into the reaction flask. The resultant suspension was refluxed at 88 °C overnight. After cooling to room temperature, the reaction mixture was filtered and the filtrate was concentrated in vacuum. The resultant residue was purified by column chromatography with DCM-PE (1:1 in volumetric ratio) as eluent to afford compound **S5** as a white solid (12.5 g, 68% yield). ^{1}H NMR (400 MHz, $CDCl_3$, 298 K): δ 7.86-7.83 (m, 2H), 7.73-7.67 (m, 2H), 3.67 (t, J = 6.9 Hz, 2H), 3.40 (t, J = 6.9 Hz, 2H), 1.90-1.78 (m, 2H), 1.74-1.60 (m, 2H), 1.45-1.36 (m, 2H), 1.35-1.24 (m, 10H).

Synthesis of S6: Mixing compound **S5** (5.5 g, 15 mmol) and 3,5-dimethoxyphenol (2.3 g, 15 mmol) in acetonitrile (100 mL), then K_2CO_3 (4.1 g, 30 mmol) was added into the reaction flask. The resultant suspension was refluxed at 88 °C overnight. After cooling to room temperature, the reaction mixture

was filtered and the filtrate was concentrated in vacuum. The resultant residue was purified by column chromatography with DCM-PE (1:2 in volumetric ratio) as eluent to afford compound **S6** as a white solid (5.2 g, 79% yield). ^{1}H NMR (300 MHz, $CDCl_3$, 298 K): δ 7.84-7.82 (m, 2H), 7.72-7.67 (m, 2H), 6.07 (s, 3H), 3.93-3.88 (t, J = 6.1 Hz, 2H), 3.76 (s, 6H), 3.71-3.65 (t, J = 7.5 Hz, 2H), 1.80-1.67 (m, 4H), 1.43-1.30 (m, 12H).

Synthesis of S7: Under N_2 atmosphere, compound **S6** (1.76 g, 4 mmol) in MeOH/THF (10/100 mL), then hydrazine hydrate (4.0 mL, 10 mmol) was added drop-wise to the solution, then the reaction mixture was refluxed at 66 °C overnight. After cooling to room temperature, the reaction mixture was filtered and the filtrate was concentrated in vacuum. The resultant residue was purified by column chromatography with DCM-CH_3OH(1:10 in volumetric ratio) as eluent to afford compound **S7** as a viscous colorless oil (0.7 g, 56% yield). ^{1}H NMR (300 MHz, $CDCl_3$, 298 K): δ 6.08 (s, 3H), 3.91 (t, J = 6.6 Hz, 2H), 3.77 (s, 6H), 2.68 (t, J = 6.9 Hz, 2H), 1.75 (q, J = 7.1 Hz, 2H), 1.30 (m, 14H).

Synthesis of S8: A $CHCl_3$ (20 mL) solution of triphosgene (196 mg, 0.66 mmol) was added into a Schlenk flask, the Schlenk flask was evacuated and back-filled with N_2 three times. Then a $CHCl_3$ (20 mL) solution of 4-ethynylbenzenamine (234 mg, 4.0 mmol) and triethylamine (1.5 mL) were added into the reaction flask and stirred at 0 °C. After 4 h, adding the $CHCl_3$ (20 mL) solution of **S7** (620 mg, 2.0 mmol) into the reaction flask, then the reaction mixture was allowed to warm to room temperature and stirred for 18 h. The resultant residue was purified by column chromatography with PE-Acetone(5:1 in volumetric ratio) as eluent to afford compound **S8** as a pale-yellow solid (650 mg, 72% yield). ^{1}H NMR (400 MHz, Acetone-d_6, 298 K): δ 8.03 (s, 1H), 7.52-7.48 (d, J = 6.0 Hz, 2H), 7.36-7.33 (d, J = 6.0 Hz, 2H), 6.07 (s, 3H), 5.83 (t, 1H), 3.95-3.92 (t, 2H), 3.74 (s, 6H), 3.49 (s, 1H), 3.21-3.18 (m, 2H), 1.75-1.72 (m, 2H), 1.50-1.28 (m, 14H).

Synthesis of S9: A round-bottom flask was charged with *cis*-5-norbornene-*exo*-2,3-dicarboxylic anhydride (8.2 g, 50 mmol) and 6-aminohexanol (5.86 g, 50 mmol). Then, 100 mL of toluene was added to the flask, followed by triethylamine (2.0 mL, 14.4 mmol). The reaction mixture was refluxed at 120°C overnight. After cooling to room temperature, the reaction mixture was concentrated in vacuum. The resultant residue was purified by column chromatography with PE-EA (1:1 in volumetric ratio) as eluent to afford compound **S9** as a colorless oil (8.2 g, 62% yield). ^{1}H NMR (400 MHz, $CDCl_3$, 298 K): 6.28 (s, 2H), 4.02 (t, J = 6.4 Hz, 2H), 3.50-3.41 (t, J = 7.6 Hz, 2H), 3.33 (t, J = 7.2 Hz, 2H),

2.68 (s, 2H), 1.68-1.50 (m, 6H), 1.47-1.38 (m, 2H).

Synthesis of S10: Under N_2 atmosphere, diisopropyl azodicarboxylate (480 µL, 2.4 mmol) was added drop-wise to a mixture of **S1** (526 mg, 2 mmol), **S9** (549 mg, 2 mmol) and triphenyl phosphine (630 mg, 2.4 mmol) in CH_2Cl_2 (20 mL) at 0 °C, then the reaction mixture was allowed to warm to room temperature and stirred overnight. The solution was concentrated and the residue was purified through column chromatography with EA-PE (1:1 in volumetric ratio) as eluent to afford compound **S10** as a viscous colorless oil (670 mg, 64% yield). ^{1}H NMR (400 MHz, $CDCl_3$, 298 K): δ 7.39 (d, J = 8.7 Hz, 2H), 6.79 (d, J = 8.7 Hz, 2H), 6.28 (s, 2H), 3.93 (t, J = 6.4 Hz, 2H), 3.50-3.40 (t, J = 7.6 Hz ,2H), 3.27 (d, J = 3.6 Hz, 2H), 2.67 (s, 2H), 1.76 (p, J = 6.7 Hz, 2H), 1.56 -1.18 (m, 8H), 1.11 (s, 21H). ^{13}C NMR (101 MHz, $CDCl_3$, 298 K): δ 178.28, 159.17, 137.95, 133.61, 115.66, 114.39, 107.29, 88.62, 67.86, 47.94, 45.29, 42.86, 38.73, 29.09, 27.82, 26.81, 25.71, 18.83, 11.47. HR-ESI-MS: Calculated for $[C_{32}H_{46}NO_3Si+H]^+$: 520.3169, Found: 520.3247.

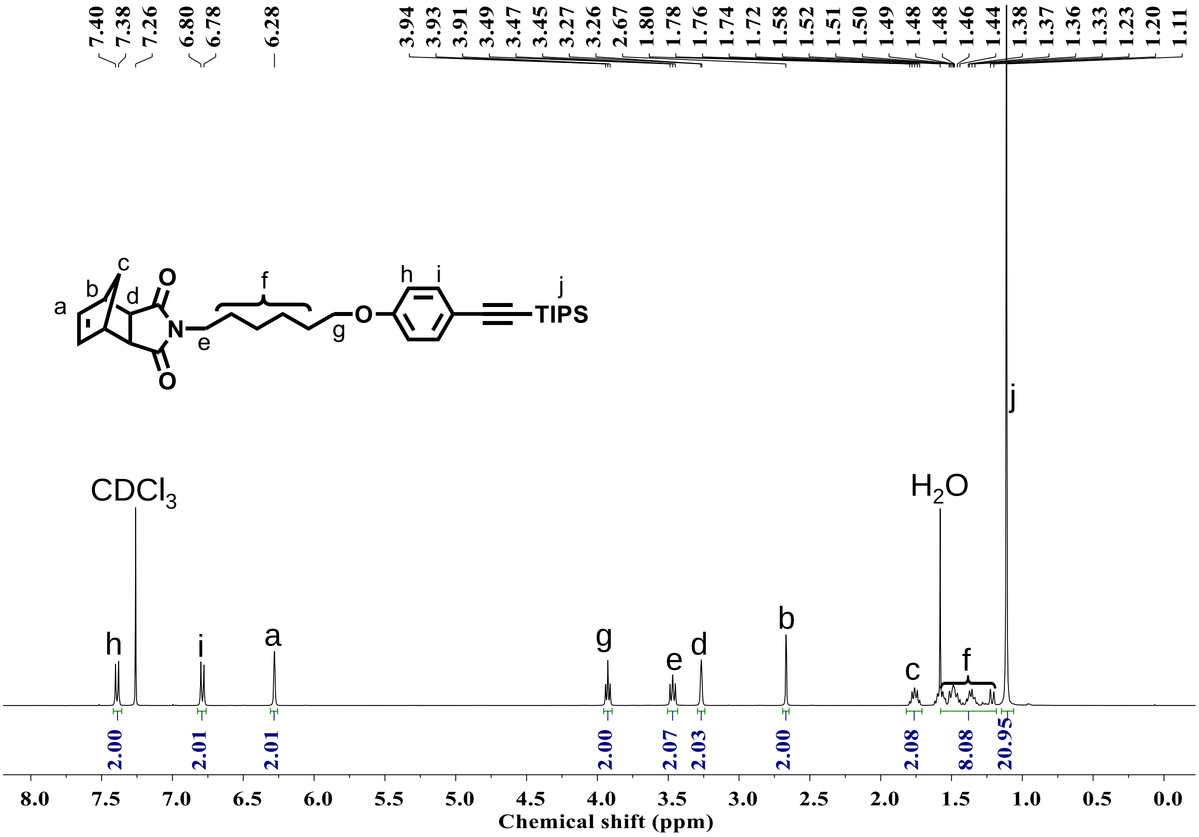

Supplementary Fig. 3 ^{1}H NMR spectrum ($CDCl_3$, 298 K, 400 MHz) of **S10**.

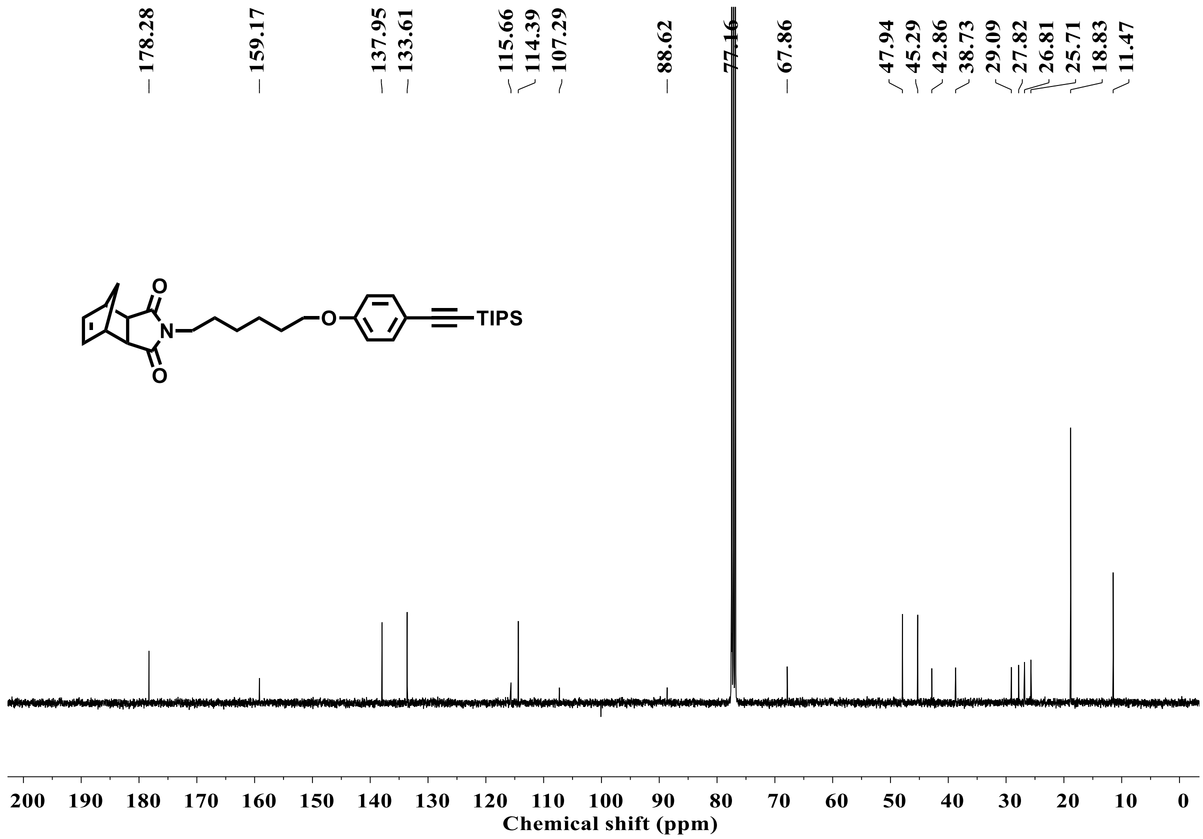

Supplementary Fig. 4 ^{13}C NMR spectrum ($CDCl_3$, 298 K, 101 MHz) of **S10**.

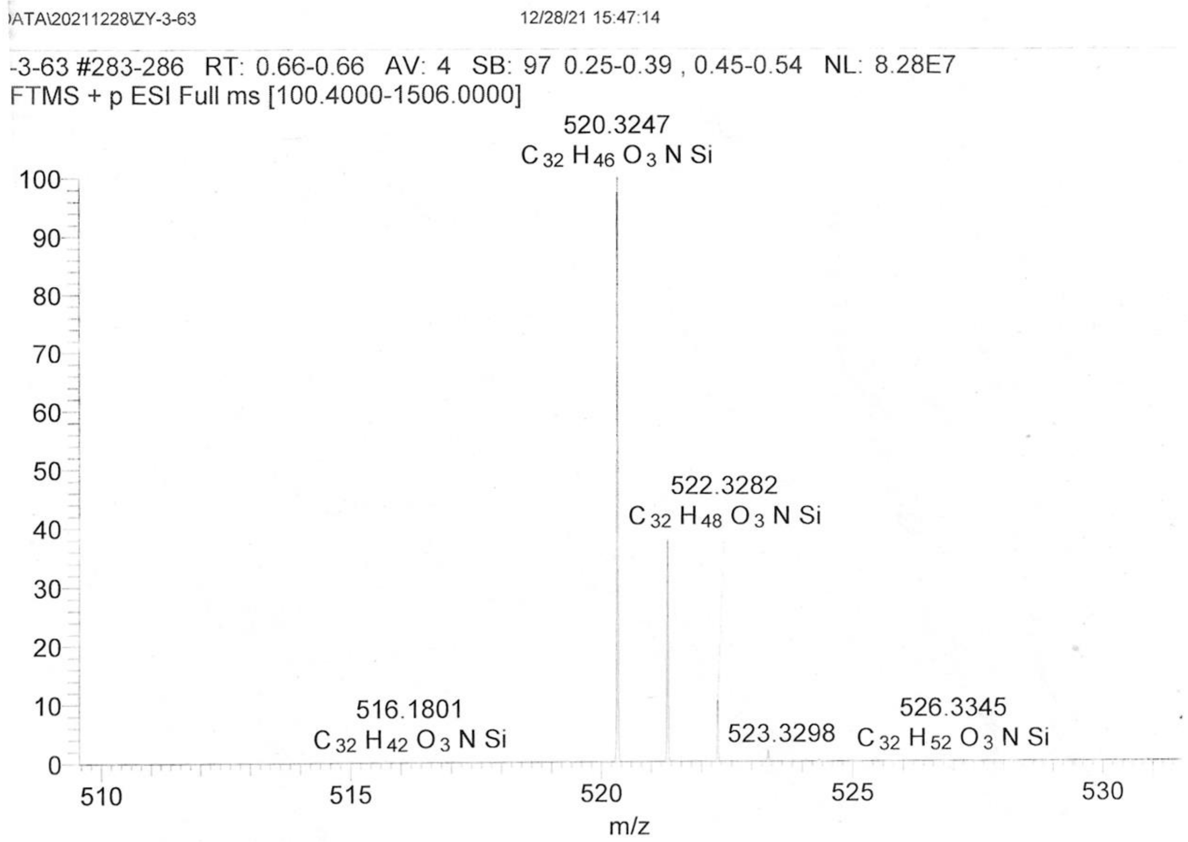

Supplementary Fig. 5 HR-MS (ESI) spectrum of **S10**.

Synthesis of 2: Tetrabutylammonium fluoride (4 mL, 3.2 mmol) was added drop-wise to a mixture of **S10** (670 mg, 1.3 mmol) at 0 °C, then the reaction mixture was allowed to warm to room temperature and stirred 30 min. the obtained residue was washed by water, then dried with Na_2SO_4 and concentrated. The residue was further purified by column chromatography with DCM-PE (1:1 in volumetric ratio) as eluent to afford compound **2** as a white solid (400 mg, 85% yield). ^{1}H NMR (400 MHz, $CDCl_3$, 298 K): δ 7.45-7.36 (d, J = 8.7 Hz, 2H), 6.87-6.77 (d, J = 8.7 Hz, 2H), 6.28 (t, J = 2.1 Hz, 2H), 4.00-3.88 (t, J = 6.3 Hz, 2H), 3.42-3.52 (m, 2H), 3.33-3.23 (t, J = 1.8 Hz, 2H), 2.98 (s, 2H), 2.69-2.63 (d, J = 1.2 Hz, 2H), 1.84-1.70 (m, 2H), 1.54-1.33 (m, 6H), 1.30-1.77 (m, 2H). ^{13}C NMR (101 MHz, $CDCl_3$, 298 K): δ 178.27, 159.52, 137.94, 133.68, 114.51, 113.98, 83.86, 75.82, 67.85, 47.93, 45.28, 42.85, 38.72, 29.07, 27.81, 26.79, 25.71. HR-ESI-MS: Calculated for $[C_{23}H_{26}NO_3+H]^+$: 364.1834, Found: 364.1898.

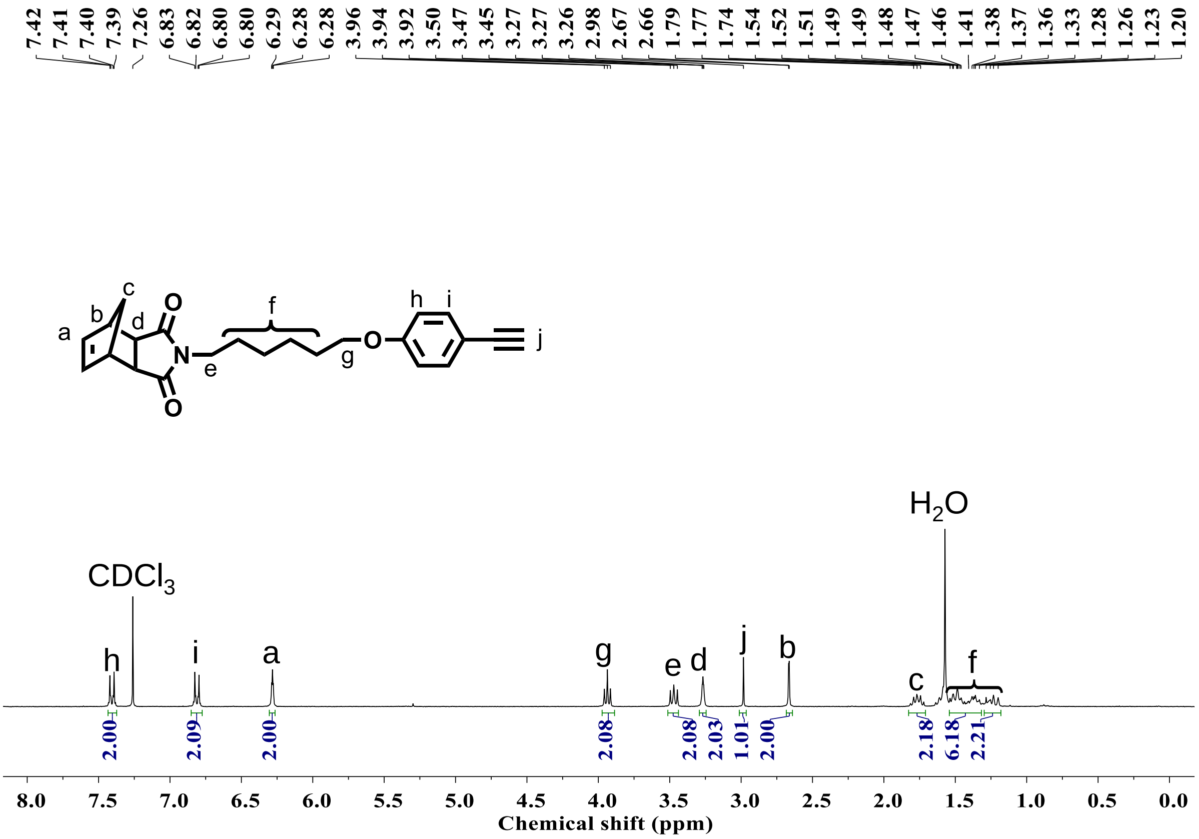

Supplementary Fig. 6 ^{1}H NMR spectrum ($CDCl_3$, 298 K, 400 MHz) of the **2**.

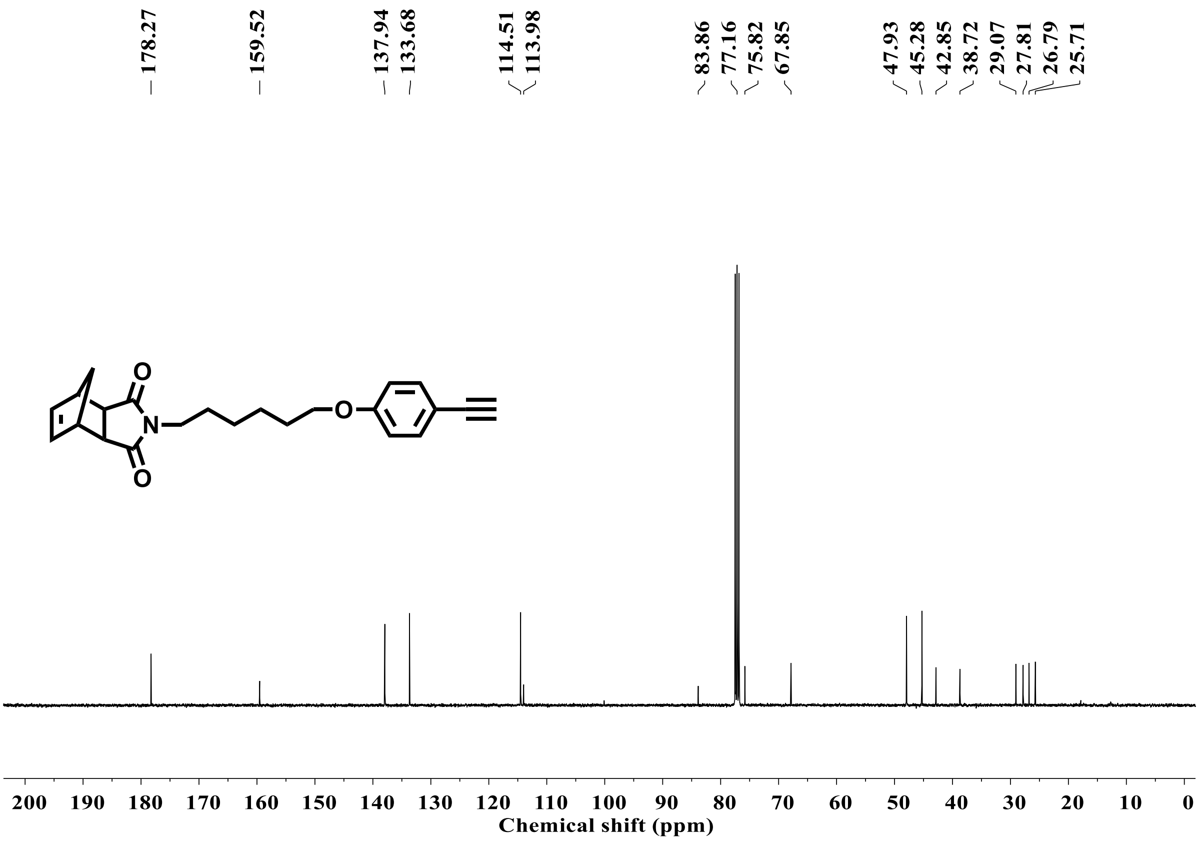

Supplementary Fig. 7 ^{13}C NMR spectrum ($CDCl_3$, 298 K, 101 MHz) of **2**.

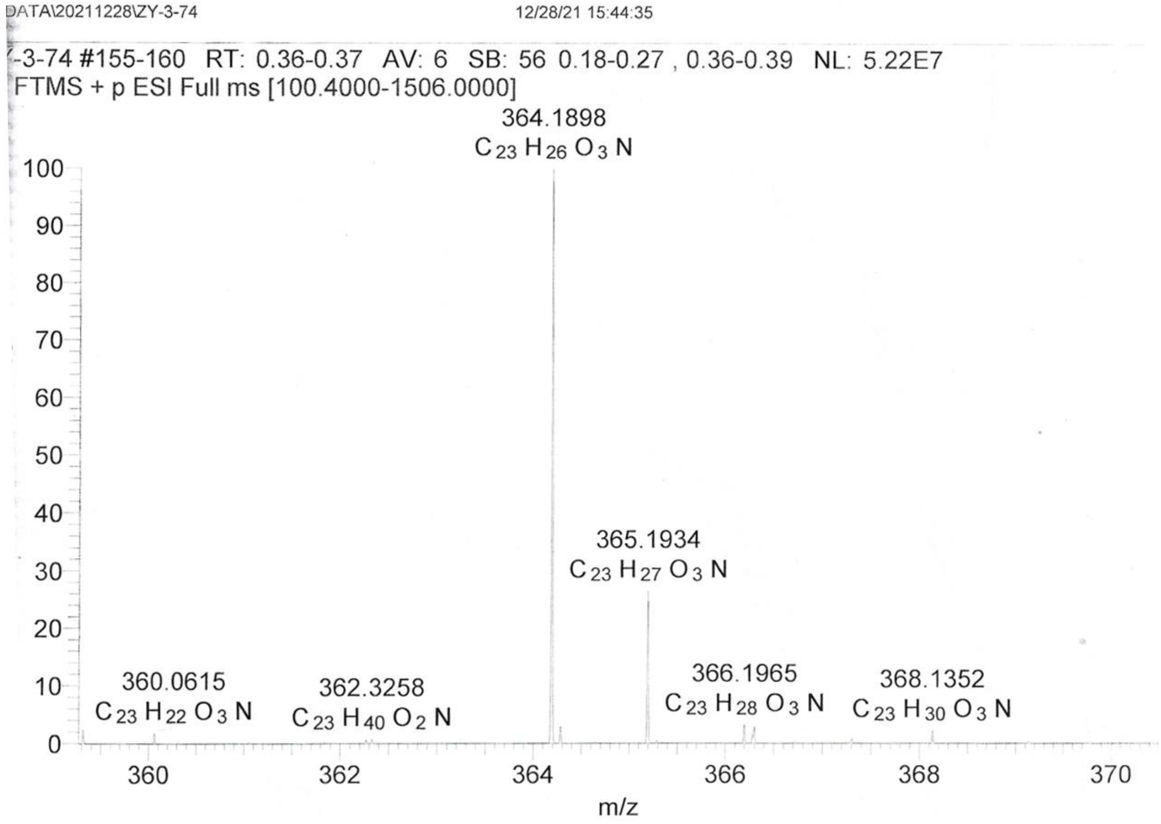

Supplementary Fig. 8 HR-MS (ESI) spectrum of **2**.

General procedure for the synthesis of rotaxane-branched dendrimer macromonomers MGn: A mixture of multiyne complexes (for **MG1**, **2**; for **MG2**, **MG1-YNE**; for **MG3, MG2-YNE**) and the [2]rotaxane **1** (for each terminal acetylene moiety, 1.1 equiv the [2]rotaxane **1** was added) in degassed dichloromethane/diethylamine (v/v, 1:1) was stirred for 12 hours at room temperature in the presence of a catalytic amount of CuI. The solvent was evaporated and the residue was purified by column chromatography and gel permeation chromatography (GPC) to afford **MGn** as pale-yellow solid.

General procedure for the synthesis of deprotected rotaxane-branched dendrimer macromonomers MGn-YNE: To a solution of **MGn** in THF, a solution of tetrabutylammonium fluoride trihydrate in THF was added dropwise. The reaction mixture was stirred at room temperature for 4 h. The obtained residue was washed by water, then dried over Na_2SO_4 and concentrated. The residue was further purified by column chromatography and gel permeation chromatography (GPC) to afford **MGn-YNE** as pale-yellow solid.

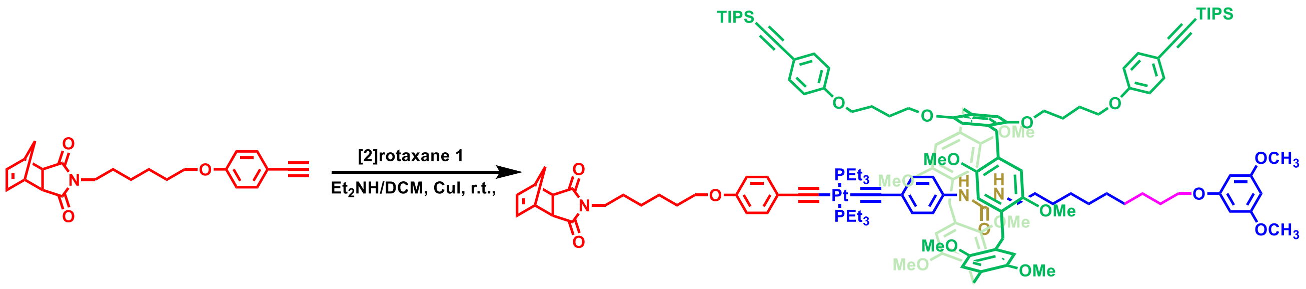

MG1

Supplementary Fig. 9 Synthesis of the first-generation rotaxane-branched dendrimer macromonomer **MG1**.

Synthesis of the first-generation rotaxane-branched dendrimer macromonomer MG1: A mixture of **2** (90 mg, 0.25 mmol) and [2]rotaxane **1** (649 mg, 0.27 mmol) were added in a Schlenk flask, the Schlenk flask was then evacuated and back-filled with N_2 three times. Next, degassed dichloromethane/diethylamine (v/v, 1:1) (10 mL) and a catalytic amount of CuI were added under an inert atmosphere. The reaction was stirred for 12 hours at room temperature. The solvent was evaporated and the residue was purified by column chromatography with DCM as eluent and gel permeation chromatography (GPC) to yield a pale-yellow solid **MG1** (520 mg, 80%). ^{1}H NMR (500 MHz, CD_2Cl_2, 298 K): δ ^{1}H NMR (500 MHz, CD_2Cl_2, 298 K): δ 7.41-7.37 (m, 4H), 7.32-7.28 (m, 2H), 7.22-7.14 (m, 4H), 6.99-6.96 (m, 4H), 6.89-6.81 (m, 10H), 6.76-6.72 (m, 2H), 6.28 (t, J = 2.0 Hz, 2H),

6.09-6.07 (m, 3H), 4.08-3.70 (m, 58H), 3.49-3.41 (t, J = 7.5 Hz, 2H), 3.22 (m, 2H), 2.83-2.76 (t, J = 4.0 Hz, 2H), 2.65 (d, J = 1.5 Hz, 2H), 2.22-2.12 (m, 12H), 2.10-1.89(m, 9H), 1.79-1.71 (m, 2H), 1.67-1.60 (m, 2H), 1.58-1.55 (m, 2H), 1.50-1.46 (m, 3H), 1.40-1.33 (m, 2H), 1.26-1.20 (m, 21H), 1.13 (s, 42H), 1.05-0.90 (m, 4H), 0.64-0.54 (m, 2H), -0.20 (m, 2H), -1.69 (m, 2H). -1.96 (m, 2H). ^{31}P NMR (202 MHz, CD_2Cl_2): δ 11.50. ^{13}C NMR (126 MHz, CD_2Cl_2): δ 178.30, 162.05, 161.49, 157.25, 151.04, 150.72, 150.69, 138.18, 133.77, 133.74, 132.15, 131.52, 129.01, 128.73, 128.68, 128.66, 119.29, 115.36, 114.78, 114.74, 114.70, 114.62, 114.51, 113.56, 113.44, 93.72, 92.82, 68.69, 68.46, 68.27, 68.22, 68.19, 68.16, 57.25, 56.82, 56.77, 56.70, 55.76, 55.69, 55.67, 48.20, 45.62, 43.06, 40.07, 38.86, 31.43, 30.82, 30.46, 30.25, 29.82, 29.51, 29.44, 29.37, 28.07, 27.12, 27.08, 27.00, 26.82, 26.71, 26.49, 26.02, 25.27, 18.87, 18.85, 16.92, 16.78, 16.64, 11.79, 11.78, 8.56, 8.51, 1.18. LRMS (MALDI-TOF-MS): Calculated for [**MG1**+H]$^+$: 2624.3; Found: 2624.2. GPC: M_w = 2.5 kDa, $Đ$ = 1.02.

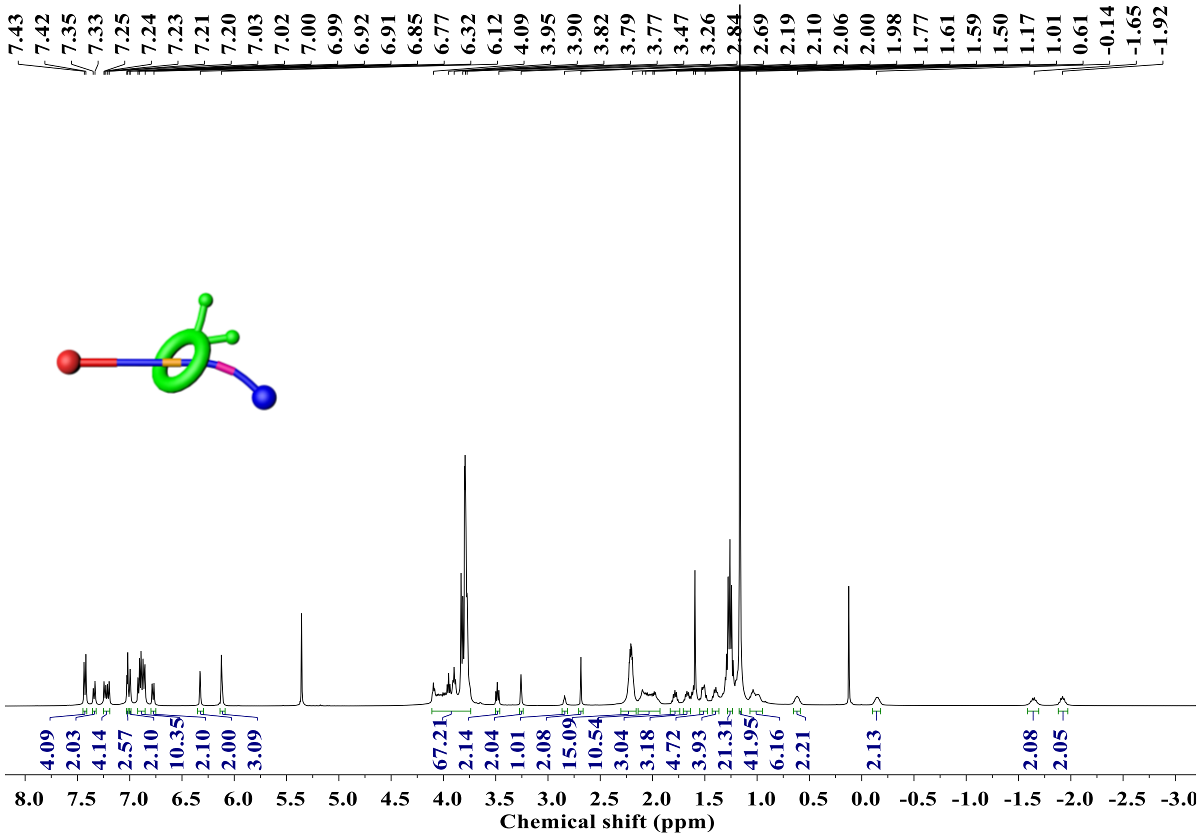

Supplementary Fig. 10 ^{1}H NMR spectrum (CD_2Cl_2, 298 K, 500 MHz) of macromonomer **MG1**.

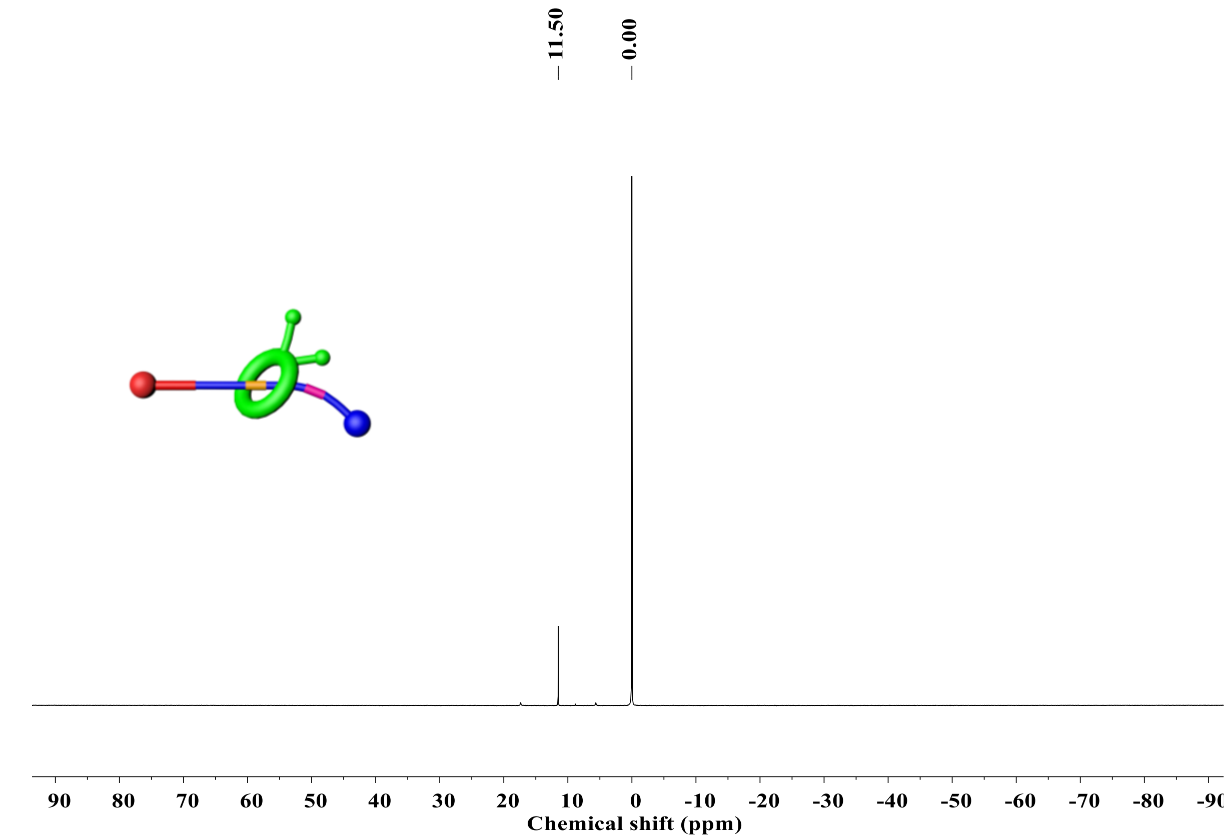

Supplementary Fig. 11 ^{31}P NMR spectrum (CD_2Cl_2, 298 K, 202 MHz) of macromonomer **MG1**.

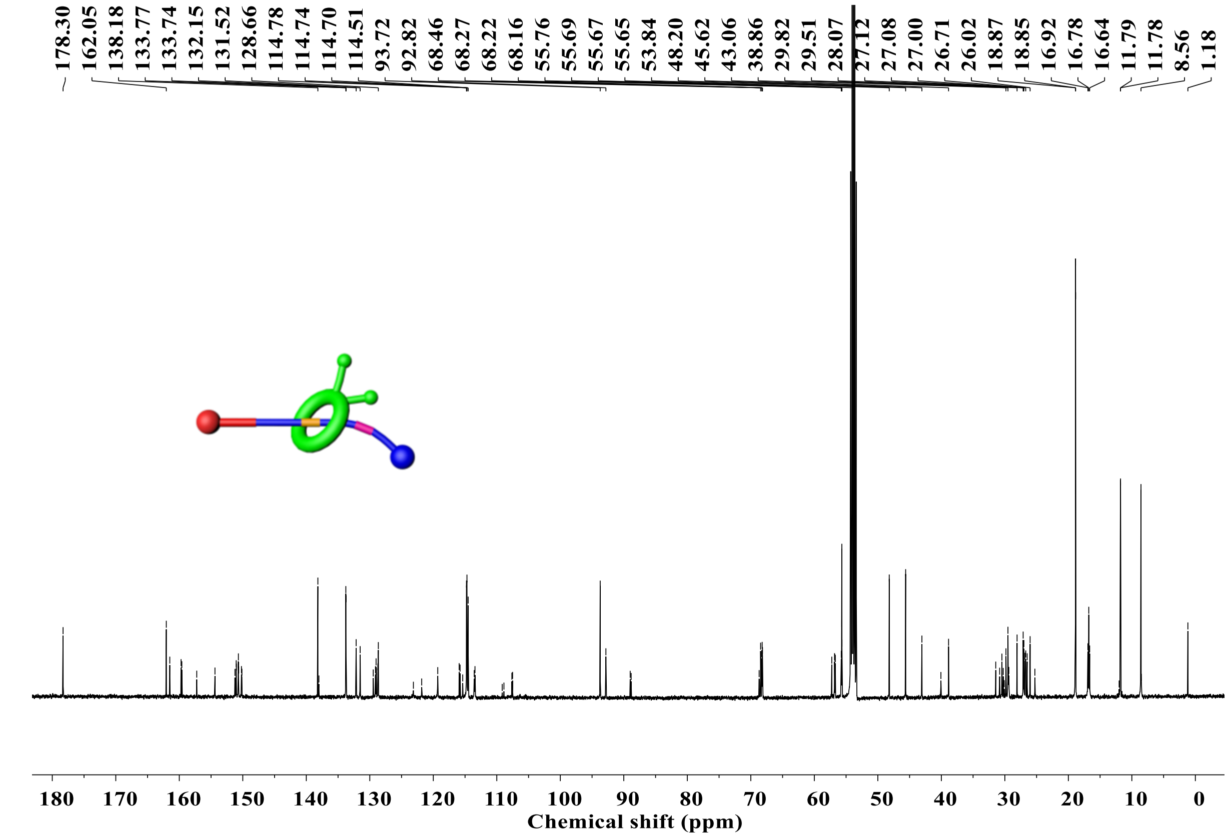

Supplementary Fig. 12 ^{13}C NMR spectrum (CD_2Cl_2, 298 K, 126 MHz) of macromonomer **MG1**.

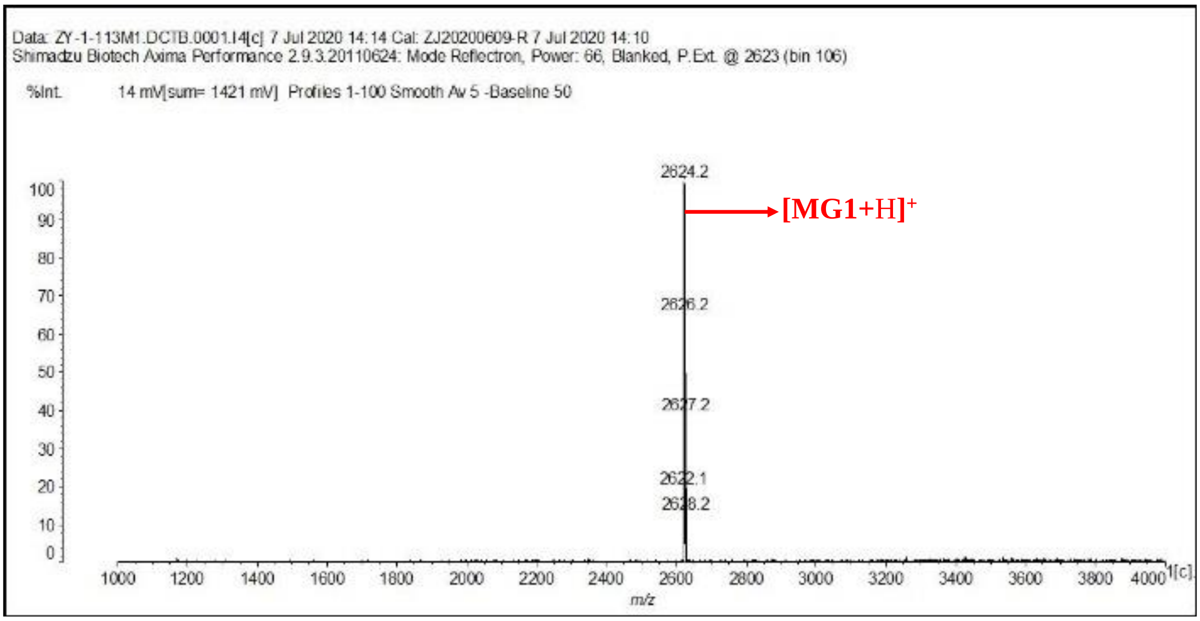

Supplementary Fig. 13 MALDI-TOF-MS spectrum of macromonomer **MG1**.

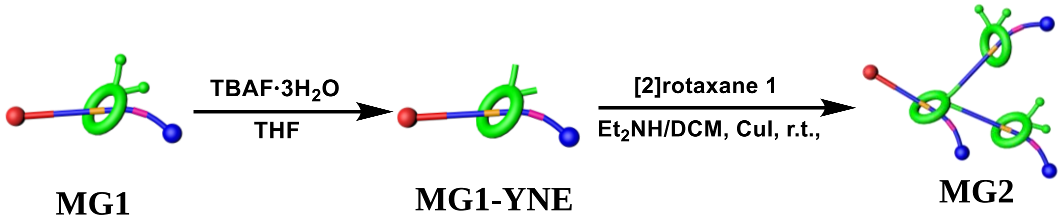

Supplementary Fig. 14 Synthesis of the second-generation rotaxane-branched dendrimer macromonomer **MG2**.

Synthesis of MG1-YNE: A solution of **MG1** (263 mg, 0.1 mmol) in THF (30 mL) and then a solution of TBAF•3H_2O (126 mg, 0.4 mmol) in THF (30 mL) was added dropwise into the reaction flask. The reaction mixture was stirred at room temperature for 4 h. The obtained residue was washed by water, then dried with Na_2SO_4 and concentrated. The solvent was evaporated and the residue was purified by column chromatography with DCM as eluent and gel permeation chromatography (GPC) to yield a pale-yellow solid **MG1-YNE** (206 mg, 91%). ^{1}H NMR (400 MHz, CD_2Cl_2, 298 K) δ 7.43-7.38 (m, 4H), 7.32-7.28 (m, 2H), 7.22-7.14 (m, 4H), 6.98 (d, J = 3.0 Hz, 3H), 6.96 (s, 2H), 6.89-6.81 (m, 10H), 6.76-6.72 (m, 2H), 6.28 (t, J = 1.9 Hz, 2H), 6.08 (s, 3H), 4.12-3.57 (m, 66H), 3.47-3.41 (m, 2H), 3.22 (m, 2H), 3.04 (d, J = 2.8 Hz, 2H), 2.84 (t, J = 4.4 Hz, 1H), 2.65 (d, J = 1.4 Hz, 2H), 2.22-2.12 (m, 14H), 2.09-1.90 (m, 10H), 1.78-1.70 (m, 3H), 1.50-1.43 (m, 5H), 1.40-1.32 (m, 3H), 1.25-1.20 (m, 25H), 1.05-0.93 (m, 5H), 0.62-0.53 (m, 2H), -0.20 (m, 2H), -1.68 (m, 2H), -1.96 (m, 2H). ^{31}P NMR (162 MHz, CD_2Cl_2, 298 K): δ 11.52. ^{13}C NMR (101 MHz, CD_2Cl_2, 298 K): δ 178.30, 162.07, 161.52,

160.04, 159.93, 157.28, 154.43, 151.24, 151.09, 150.72, 150.25, 150.16, 138.19, 133.94, 133.91, 132.16, 131.54, 129.44, 129.17, 129.05, 128.96, 128.70, 119.30, 115.31, 114.93, 114.83, 114.55, 114.39, 114.23, 113.49, 93.77, 92.85, 84.04, 83.91, 76.05, 75.97, 68.68, 68.49, 68.31, 68.24, 57.20, 56.85, 56.73, 55.78, 48.22, 45.64, 43.07, 40.10, 38.87, 31.44, 30.81, 30.47, 30.26, 30.11, 29.84, 29.52, 28.08, 27.12, 26.81, 26.70, 26.49, 26.03, 25.30, 16.81, 8.57. LRMS (MALDI-TOF-MS): Calculated for [**MG1-YNE**+H]$^{+}$: 2312.1; Found: 2312.2.

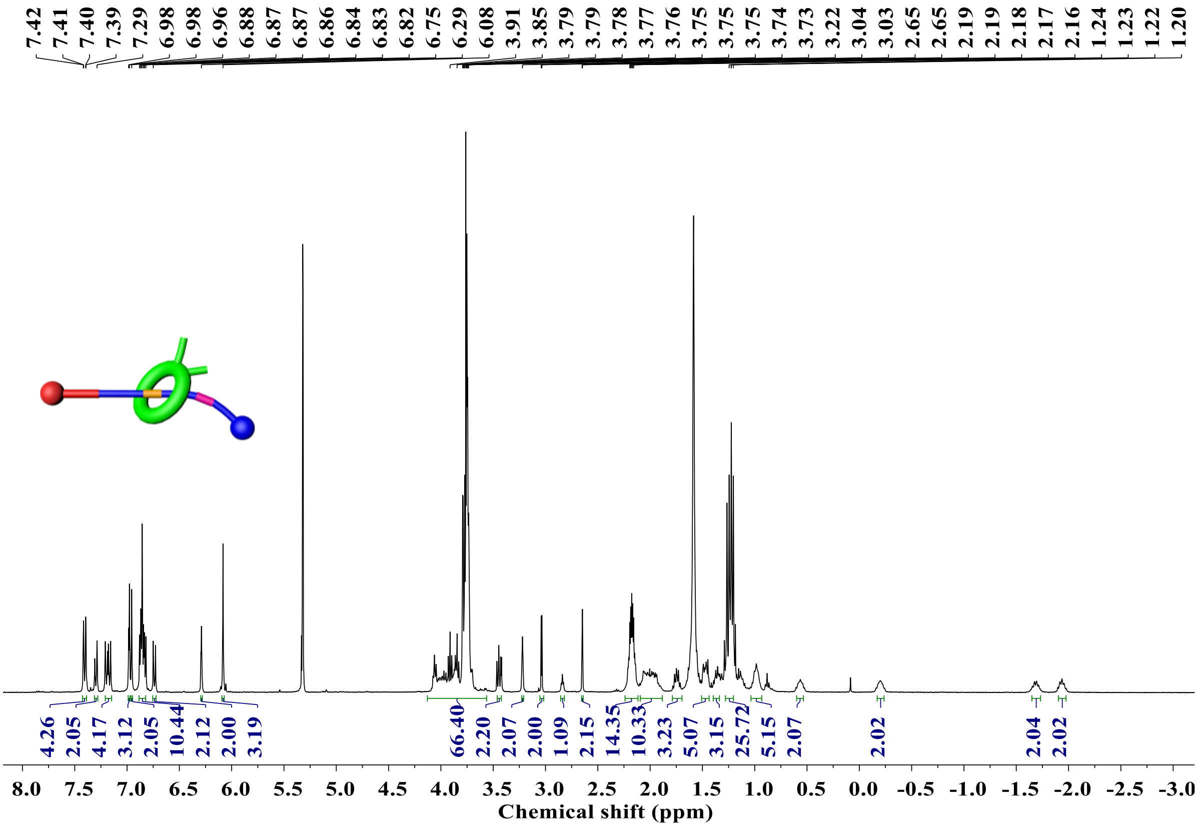

Supplementary Fig. 15 ^{1}H NMR spectrum (CD_2Cl_2, 298 K, 400 MHz) of **MG1-YNE**.

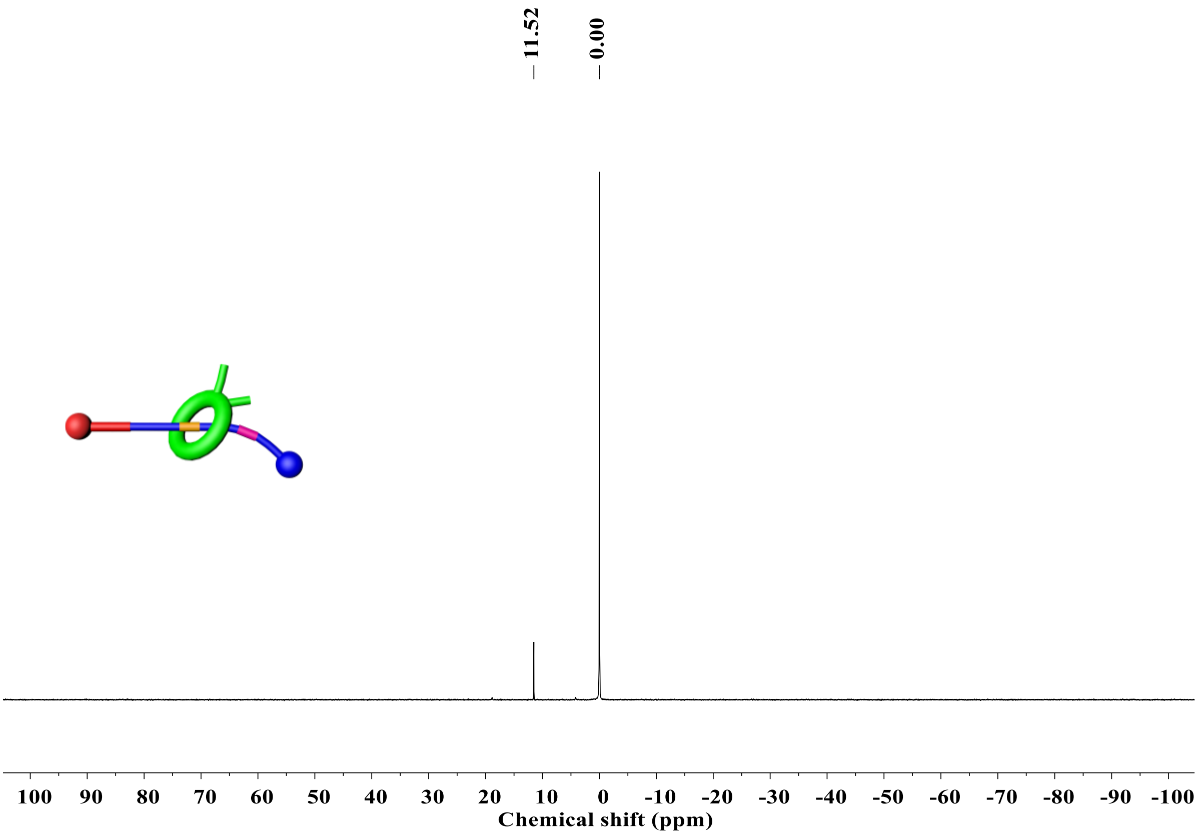

Supplementary Fig. 16 ^{31}P NMR spectrum (CD_2Cl_2, 298 K, 162 MHz) of **MG1-YNE**.

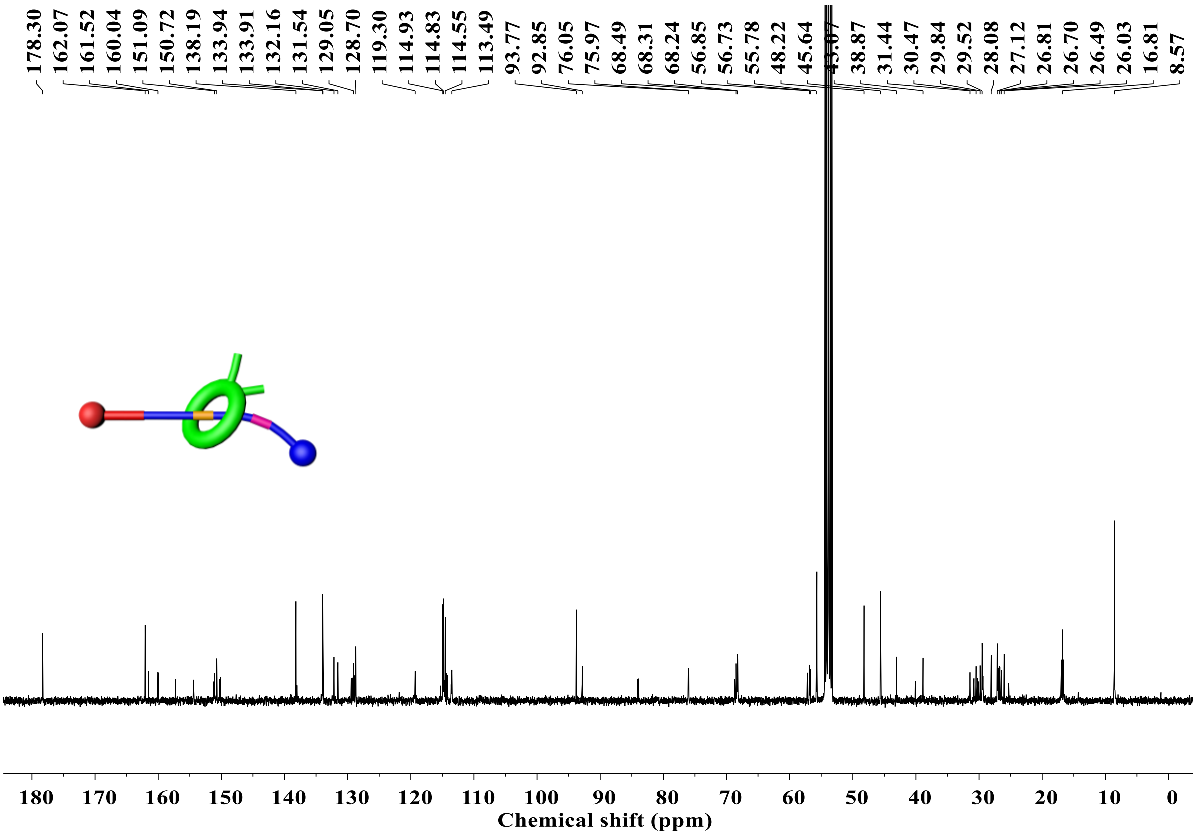

Supplementary Fig. 17 ^{13}C NMR spectrum (CD_2Cl_2, 298 K, 101 MHz) of **MG1-YNE**.

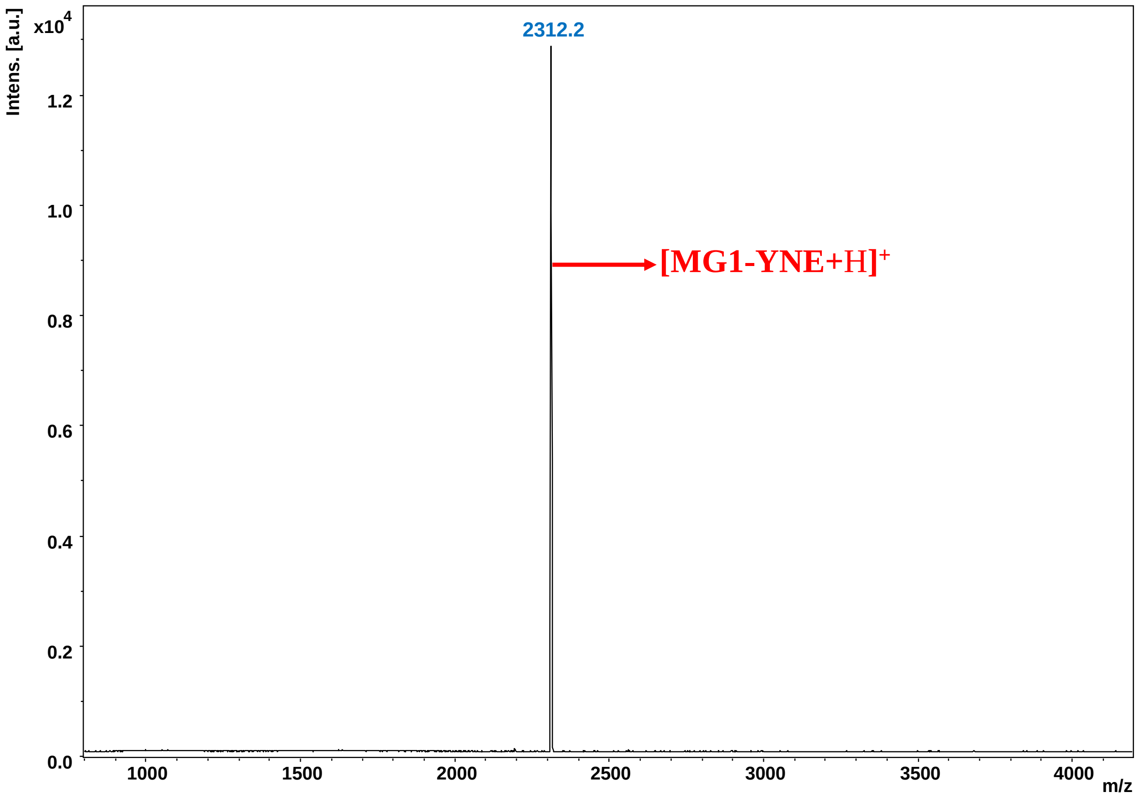

Supplementary Fig. 18 MALDI-TOF-MS spectrum of **MG1-YNE**.

Synthesis of the second-generation rotaxane-branched dendrimer macromonomer MG2:

The obtained **MG1-YNE** (206 mg, 0.089 mmol) and [2]rotaxane **1** (468 mg, 0.196 mmol) were added in a Schlenk flask, the Schlenk flask was then evacuated and back-filled with N_2 three times. Next, degassed dichloromethane/diethylamine (v/v, 1:1) (20 mL) and a catalytic amount of CuI were added under an inert atmosphere. The reaction was stirred for 12 hours at room temperature. The solvent was evaporated and the residue was purified by column chromatography with DCM/EA as eluent and gel permeation chromatography (GPC) to yield a pale-yellow solid **MG2** (435 mg, 78%). ^{1}H NMR (500 MHz, CD_2Cl_2, 298 K): δ 7.39 (d, J = 8.5 Hz, 9H), 7.31 (t, J = 8.5 Hz, 7H), 7.19 (m, 14H), 7.00-6.93 (m, 20H), 6.92-6.80 (m, 32H), 6.79-6.72 (m, 8H), 6.28 (t, J = 1.8 Hz, 2H), 6.11-6.07 (m, 9H), 4.08-3.67 (m, 206H), 3.44 (t, J = 7.5 Hz, 2H), 3.25-3.18 (t, J = 1.5 Hz, 2H), 2.95-2.88 (t, J = 4.5 Hz, 1H), 2.83-2.77 (q, J = 3.5 Hz, 2H), 2.66-2.62 (d, J = 1.0 Hz, 2H), 2.26-2.17 (m, 48H), 2.08-1.92 (m, 32H), 1.78-1.71 (m, 2H), 1.65-1.60 (m, 6H), 1.51-1.44 (m, 6H), 1.38-1.33 (m, 6H), 1.30-1.08 (m, 200H), 1.07-0.92 (m, 18H) 0.59 (d, J = 10.8 Hz, 1H), -0.19 (m, 6H), -1.54 (m, 6H), -1.68 (m, 6H), -1.60 (m, 8H), -1.95 (m, 4H). ^{31}P NMR (202 MHz, CD_2Cl_2, 298 K): δ 11.84, 11.81. ^{13}C NMR (126 MHz, CD_2Cl_2, 298 K): δ 178.29, 162.05, 161.49, 159.72, 159.60, 154.38, 151.24, 151.03, 150.72, 150.69, 150.64,

150.21, 138.17, 138.01, 133.77, 133.74, 132.20, 132.15, 131.52, 129.46, 129.11, 129.01, 128.95, 128.73, 128.69, 128.66, 123.12, 119.31, 115.36, 114.78, 114.74, 114.70, 114.62, 114.56, 114.52, 114.49, 113.56, 113.44, 109.19, 93.74, 93.72, 92.82, 92.79, 89.00, 68.68, 68.50, 68.46, 68.27, 68.21, 68.18, 68.16, 68.09, 57.25, 56.81, 56.77, 56.70, 55.76, 55.73, 55.69, 55.67, 54.10, 53.69, 48.20, 45.62, 43.06, 40.07, 38.86, 31.43, 30.82, 30.46, 30.40, 30.32, 30.25, 29.82, 29.78, 29.51, 29.38, 28.07, 27.16, 27.12, 27.09, 27.00, 26.82, 26.70, 26.63, 26.53, 26.49, 26.02, 25.27, 18.87, 18.85, 16.93, 16.84, 16.79, 16.66, 11.79, 11.78, 8.57, 1.18. LRMS (MALDI-TOF-MS): Calculated for [**MG2**+Li]$^+$: 6843.6; Found: 6842.6. GPC: M_w = 6.8 kDa, $Đ$ = 1.02.

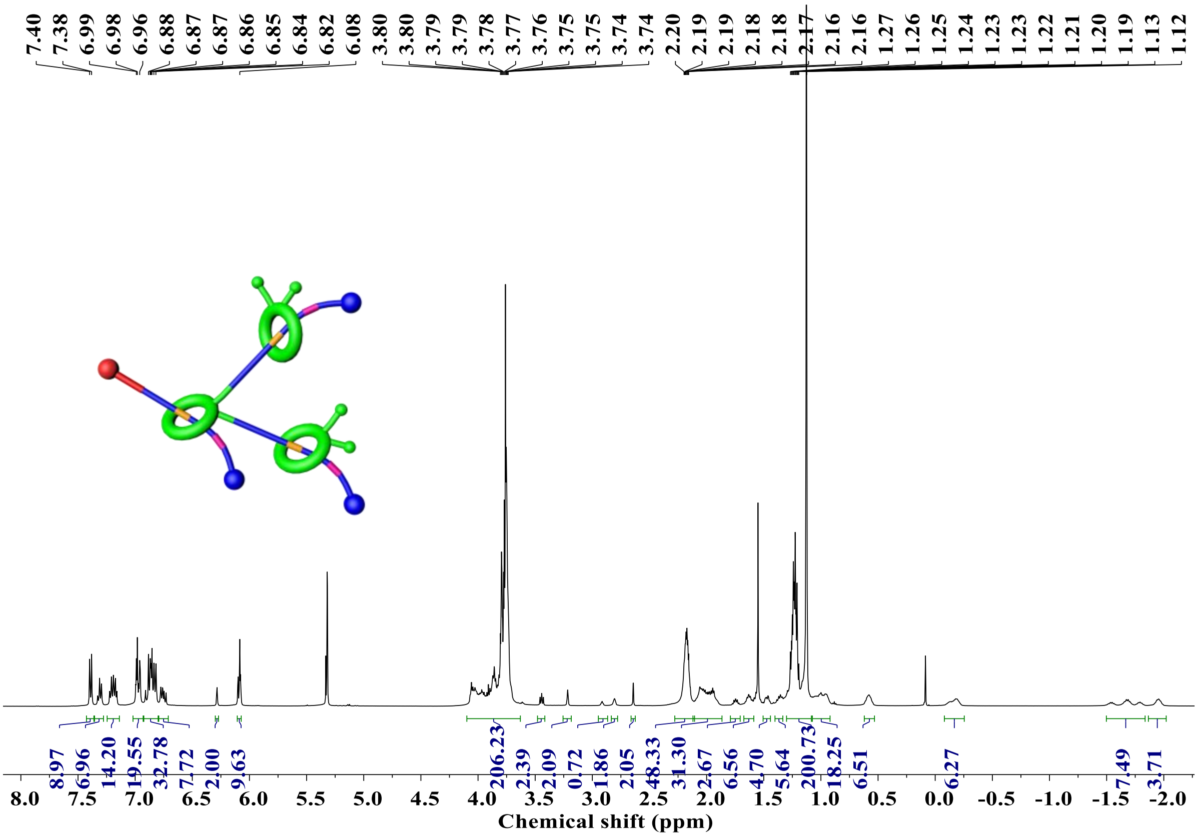

Supplementary Fig. 19 ^{1}H NMR spectrum (CD_2Cl_2, 298 K, 500 MHz) of macromonomer **MG2**.

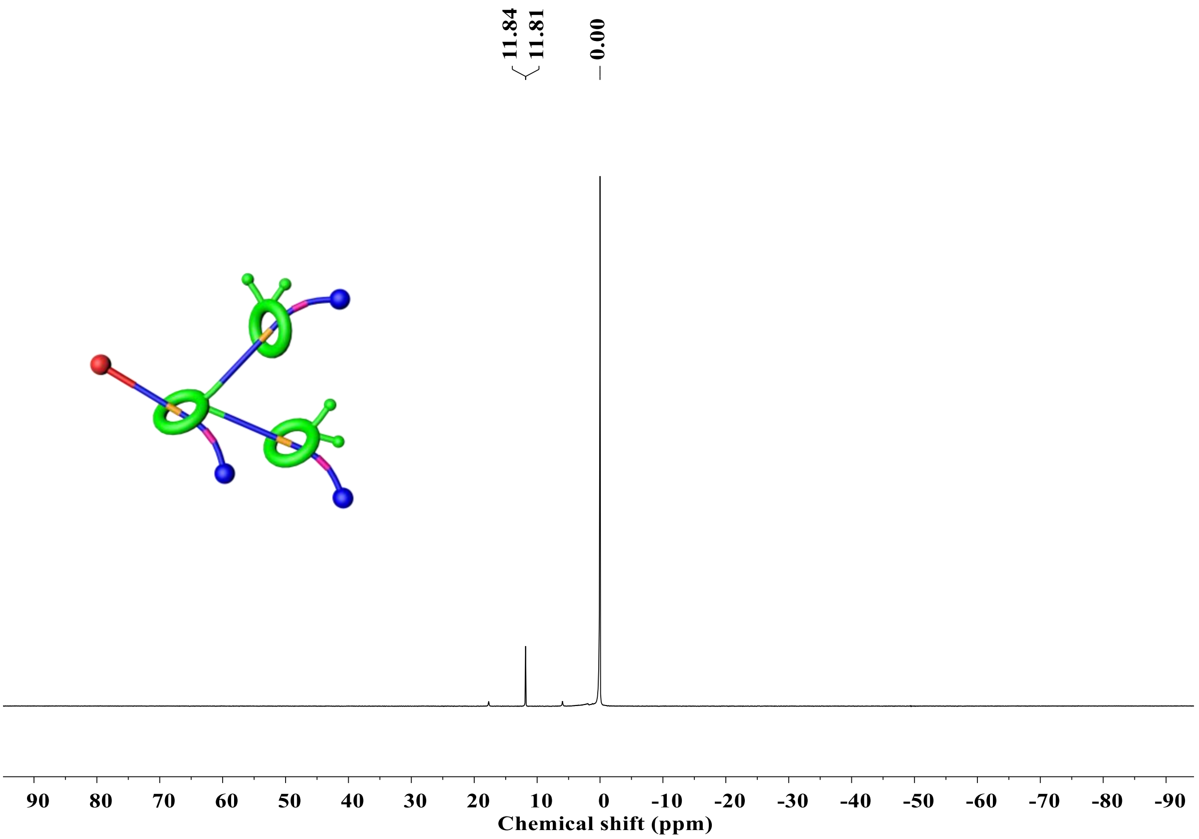

Supplementary Fig. 20 ^{31}P NMR spectrum (CD_2Cl_2, 298 K, 202 MHz) of the macromonomer **MG2**.

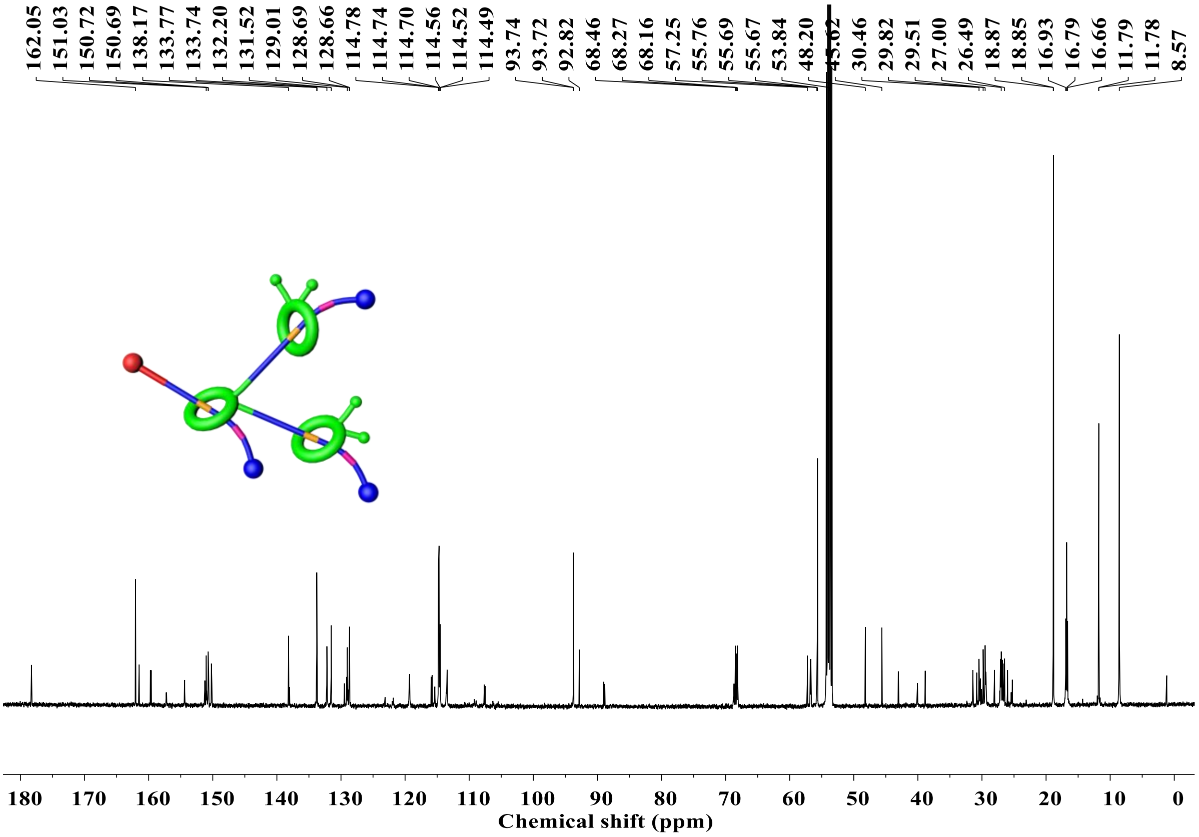

Supplementary Fig. 21 ^{13}C NMR spectrum (CD_2Cl_2, 298 K, 126 MHz) of macromonomer **MG2**.

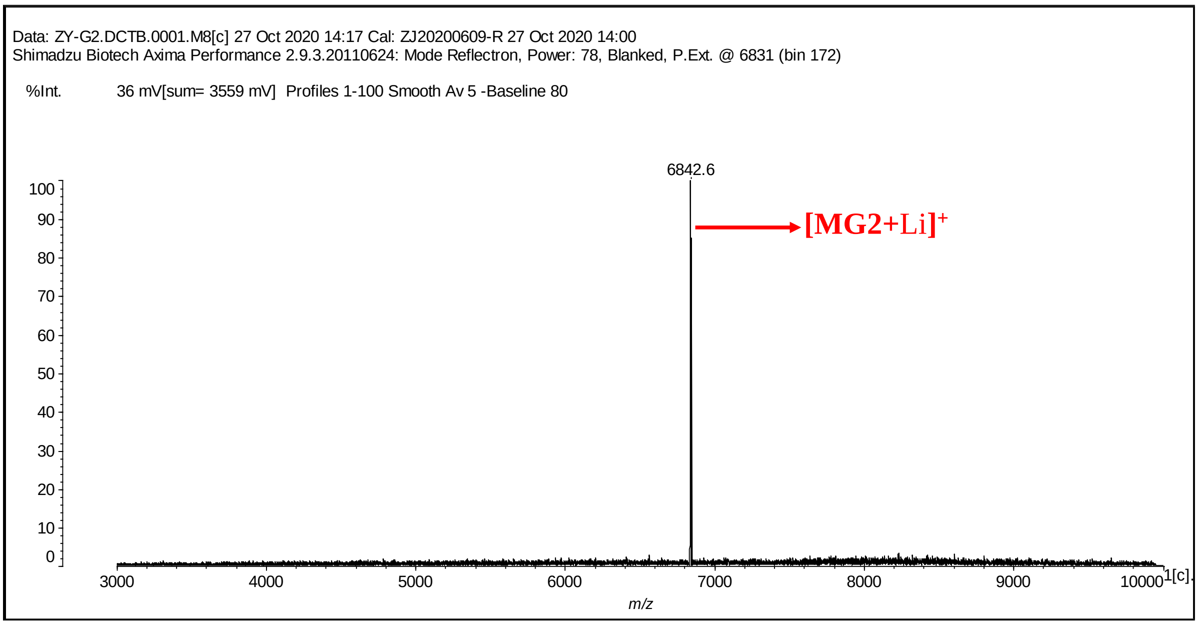

Supplementary Fig. 22 MALDI-TOF-MS spectrum of macromonomer **MG2**.

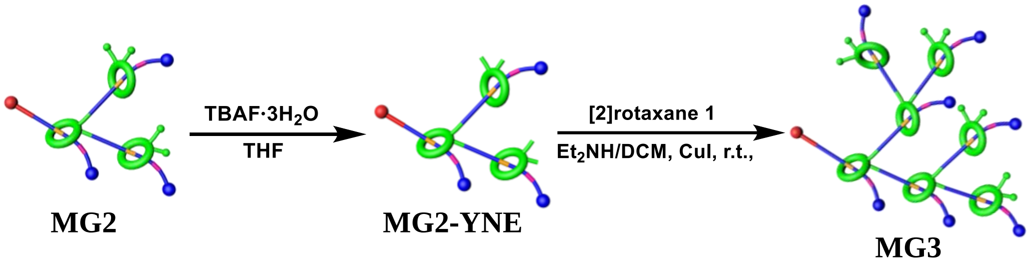

Supplementary Fig. 23 Synthesis of the third-generation rotaxane-branched dendrimer macromonomer **MG3**.

Synthesis of MG2-YNE: A solution of **MG2** (440 mg, 0.065 mmol) in THF (50 mL) and then a solution of tetrabutylammonium fluoride trihydrate (164 mg, 0.52 mmol) in THF (50 mL) was added dropwise into the reaction flask. The reaction mixture was stirred at room temperature for 4h. The obtained residue was washed by water, then dried with Na_2SO_4 and concentrated. The solvent was evaporated and the residue was purified by column chromatography with DCM/EA as eluent and gel permeation chromatography (GPC) to yield a pale-yellow solid **MG2-YNE** (320 mg, 88%). ^{1}H NMR (400 MHz, CD_2Cl_2, 298 K) δ 7.44-7.38 (m, 9H), 7.31 (dd, J = 8.6, 6.6 Hz, 9H), 7.19 (m, 14H), 7.04-6.94 (m, 19H), 6.91-6.79 (m, 32H), 6.78-6.70 (m, 7H), 6.28 (t, J = 1.9 Hz, 2H), 6.10-6.07 (m, 9H), 4.09-3.58 (m, 189H), 3.44 (t, J = 7.2 Hz, 1H), 3.22 (t, J = 1.8 Hz, 1H), 3.04 (d, J = 2.6 Hz, 2H), 2.92 (s, 2H), 2.83-2.76 (d, J = 4.7 Hz, 2H), 2.64 (d, J = 1.4 Hz, 2H), 2.27-2.16 (m, 44H), 2.10-1.88 (m, 32H), 1.80-1.71 (m, 4H), 1.68-1.60 (m, 6H), 1.31-1.07 (m, 84H), 0.98 (m, 15H), 0.57 (m, 6H), -0.16

(m, 6H), -1.56--1.83 (m,8H), -1.94 (m, 4H). ^{31}P NMR (162 MHz, CD_2Cl_2, 298 K): δ 11.50, 11.46. ^{13}C NMR (126 MHz, CD_2Cl_2, 298 K): δ 178.29, 162.07, 161.51, 160.03, 159.92, 157.27, 154.42, 151.23, 151.07, 151.04, 150.75, 150.72, 150.68, 150.24, 150.15, 138.18, 138.03, 133.94, 133.91, 132.20, 131.53, 129.43, 129.17, 129.05, 128.96, 128.75, 128.72, 128.70, 119.30, 115.30, 114.93, 114.83, 114.54, 114.39, 114.23, 113.49, 93.77, 92.84, 84.03, 83.91, 76.07, 76.00, 68.68, 68.49, 68.31, 68.22, 57.19, 56.85, 56.72, 55.78, 55.68, 48.21, 45.63, 43.06, 40.10, 38.87, 31.44, 30.81, 30.47, 30.26, 30.10, 29.81, 29.52, 28.08, 27.11, 26.99, 26.81, 26.70, 26.48, 26.02, 25.48, 25.30, 16.82, 8.58. LRMS (MALDI-TOF-MS): Calculated for [**MG2-YNE**+H]$^+$: 6211.3; Found: 6211.3.

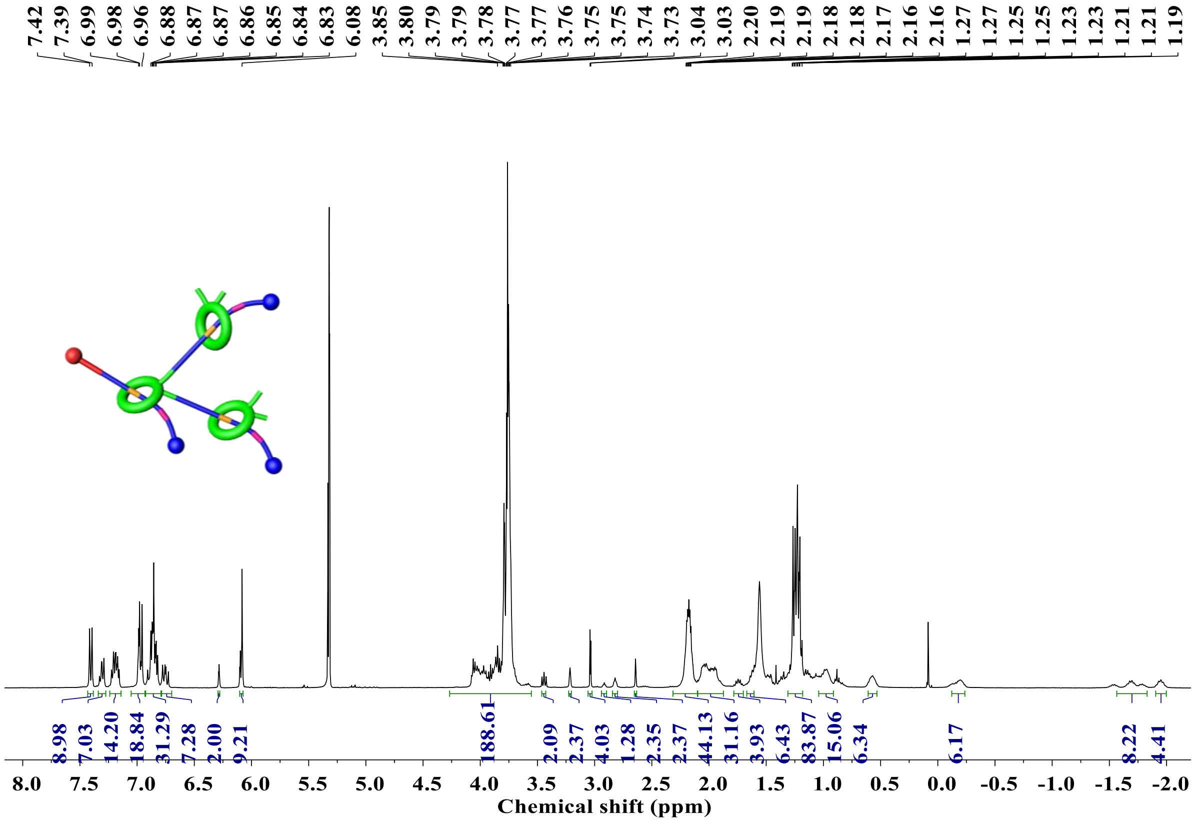

Supplementary Fig. 24 ^{1}H NMR spectrum (CD_2Cl_2, 298 K,400 MHz) of **MG2-YNE**.

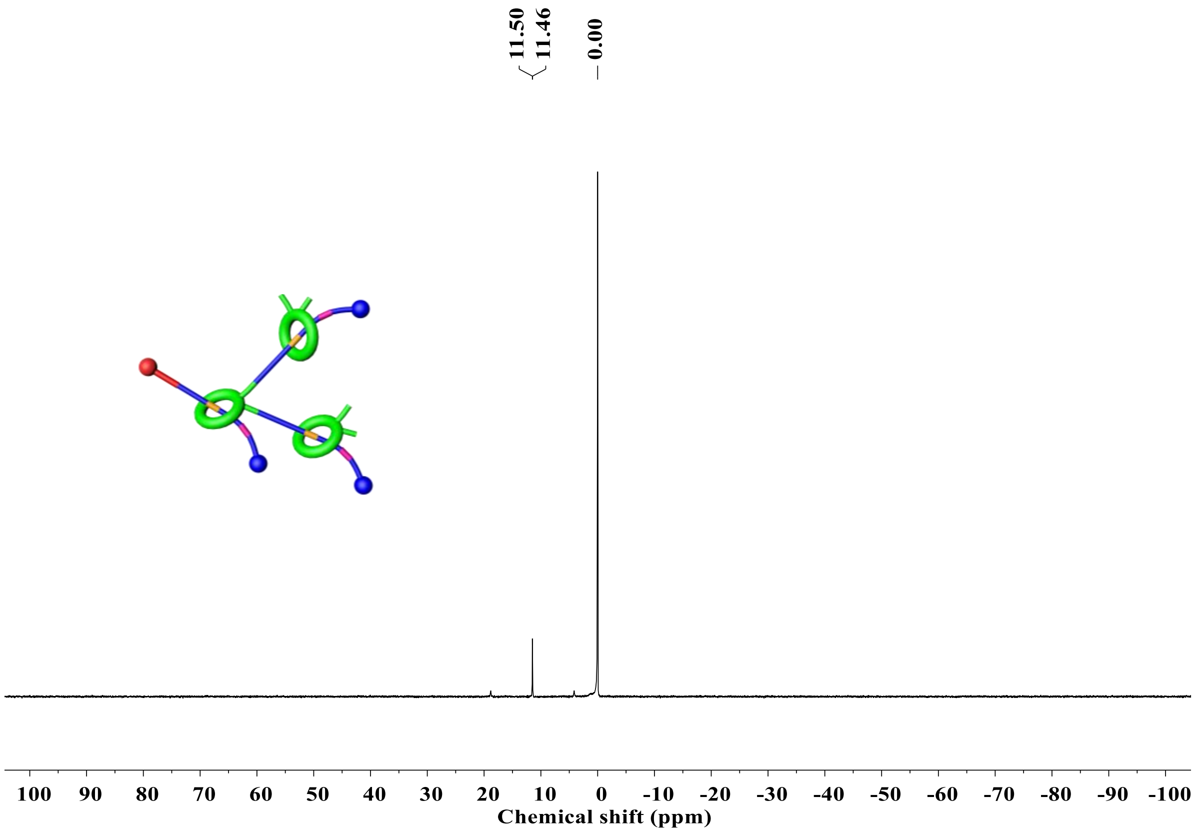

Supplementary Fig. 25 ^{31}P NMR spectrum (CD_2Cl_2, 298 K, 162 MHz) of **MG2-YNE**.

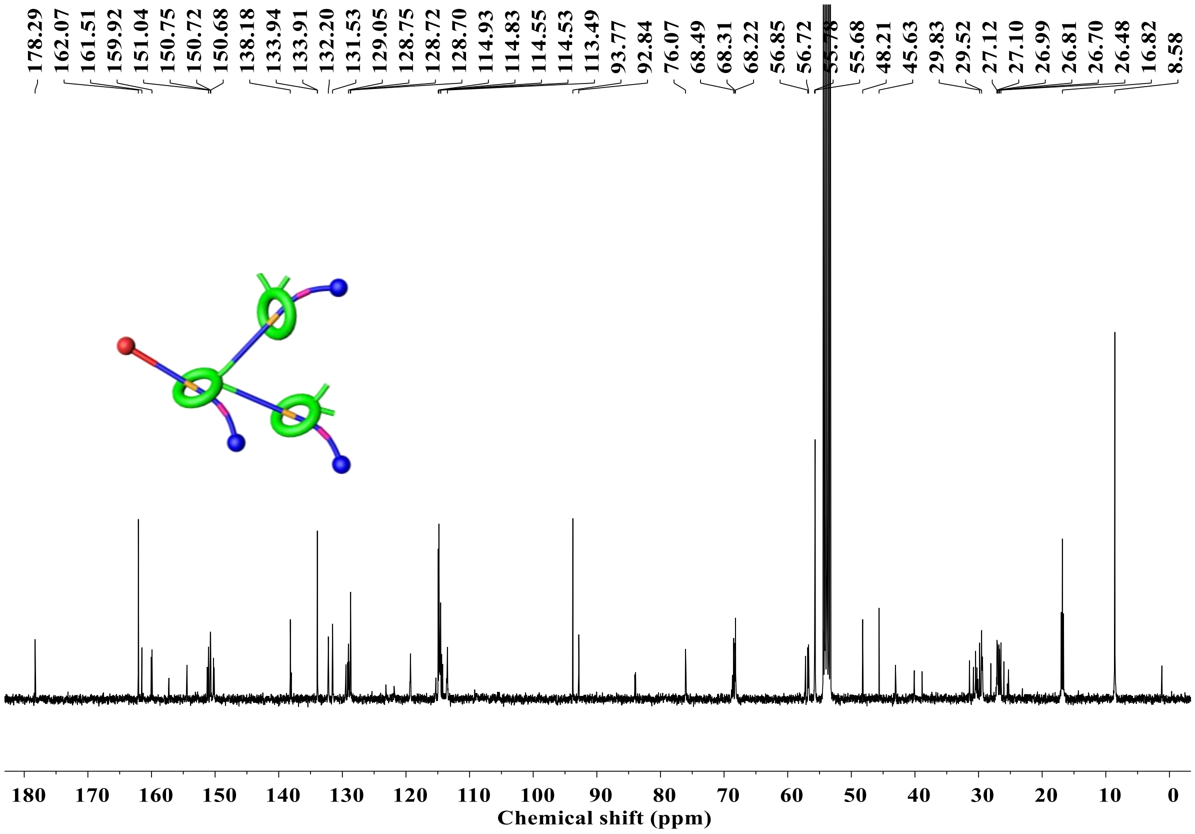

Supplementary Fig. 26 ^{13}C NMR spectrum (CD_2Cl_2, 298 K, 101 MHz) of **MG2-YNE**.

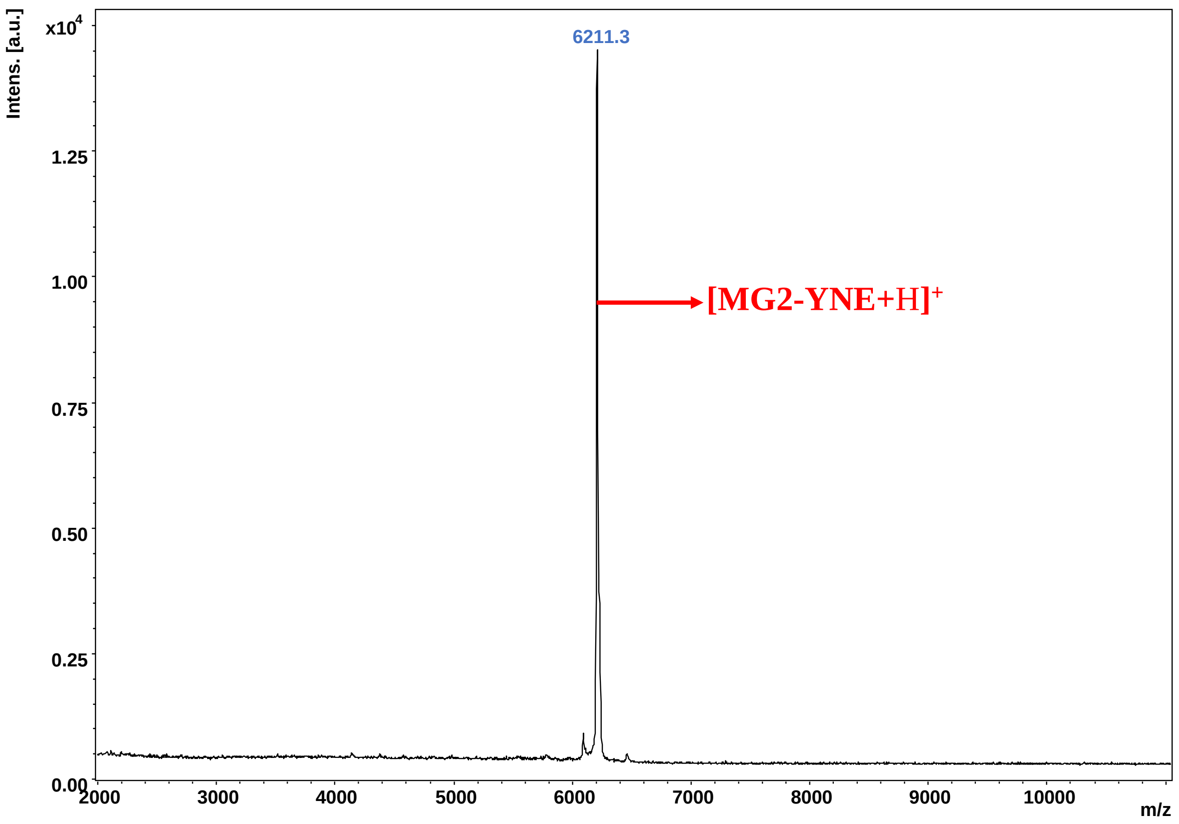

Supplementary Fig. 27 MALDI-TOF-MS spectrum of **MG2-YNE**.

Synthesis of the third-generation rotaxane-branched dendrimer macromonomer MG3: The obtained **MG2-YNE** (320 mg, 0.057 mmol) and [2]rotaxane **1** (602 mg, 0.252 mmol) were added in a Schlenk flask, the Schlenk flask was then evacuated and back-filled with N_2 three times. Next, degassed dichloromethane/diethylamine (v/v, 1:1) (20 mL) and a catalytic amount of CuI were added under an inert atmosphere. The reaction was stirred for 12 hours at room temperature. The solvent was evaporated and the residue was purified by column chromatography with DCM/EA as eluent and gel permeation chromatography (GPC) to yield a pale-yellow solid **MG3** (500 mg, 65%). ^{1}H NMR (500 MHz, CD_2Cl_2, 298 K): δ 7.54-6.60 (m, 235H), 6.28 (t, J = 1.8 Hz, 2H), 6.10-6.07 (m, 21H), 4.21-3.57 (m, 533H), 3.46 (t, J = 7.5 Hz, 2H), 3.27-3.20 (t, J = 1.5 Hz, 2H), 2.95-2.88 (t, J = 4.5 Hz, 2H), 2.83-2.77 (q, J = 3.5 Hz, 2H), 2.66-2.62 (d, J = 1.0 Hz, 2H), 2.19 (m, 205H), 1.79-1.70 (m,), 1.66-1.63 (m, 13H), 1.50-1.31(m, 24H), 1.31-1.16 (m, 232H), 1.13 (s, 230H), 1,08-0.90 (m, 56H), 0.58 (s, 18H), -0.15 (m, 18H), -1.53 (m, 6H), -1.72 (m, 16H), -1.95 (m, 9H). ^{31}P NMR (202 MHz, CD_2Cl_2, 298 K): δ 11.77. ^{13}C NMR (126 MHz, CD_2Cl_2, 298 K): δ 178.29, 162.05, 154.45, 151.25, 151.04, 150.72, 150.69, 150.64, 150.21, 138.17, 138.03, 133.77, 133.74, 132.20, 131.52, 129.47, 129.01, 128.68, 128.66, 119.34, 115.35, 114.78, 114.74, 114.70, 114.57, 114.49, 113.46, 93.74, 93.72, 92.82, 68.68, 68.50,

68.46, 68.27, 68.17, 57.24, 56.81, 56.77, 56.70, 55.79, 55.76, 55.73, 55.67, 54.11, 53.70, 48.20, 40.07, 31.43, 30.82, 30.46, 30.40, 30.25, 30.10, 29.82, 29.77, 29.51, 29.38, 27.09, 27.00, 26.82, 26.71, 26.49, 25.45, 25.27, 18.87, 18.85, 16.94, 16.80, 16.66, 11.79, 11.78, 8.58, 1.18. LRMS (MALDI-TOF-MS): Calculated for [**MG3**+H]$^{+}$: 15248.4 and [**MG3**+2H]$^{2+}$: 7624.7; Found: 15232.3 and 7630.5. GPC: M_{w} = 15.2 kDa, $Đ$ = 1.02.

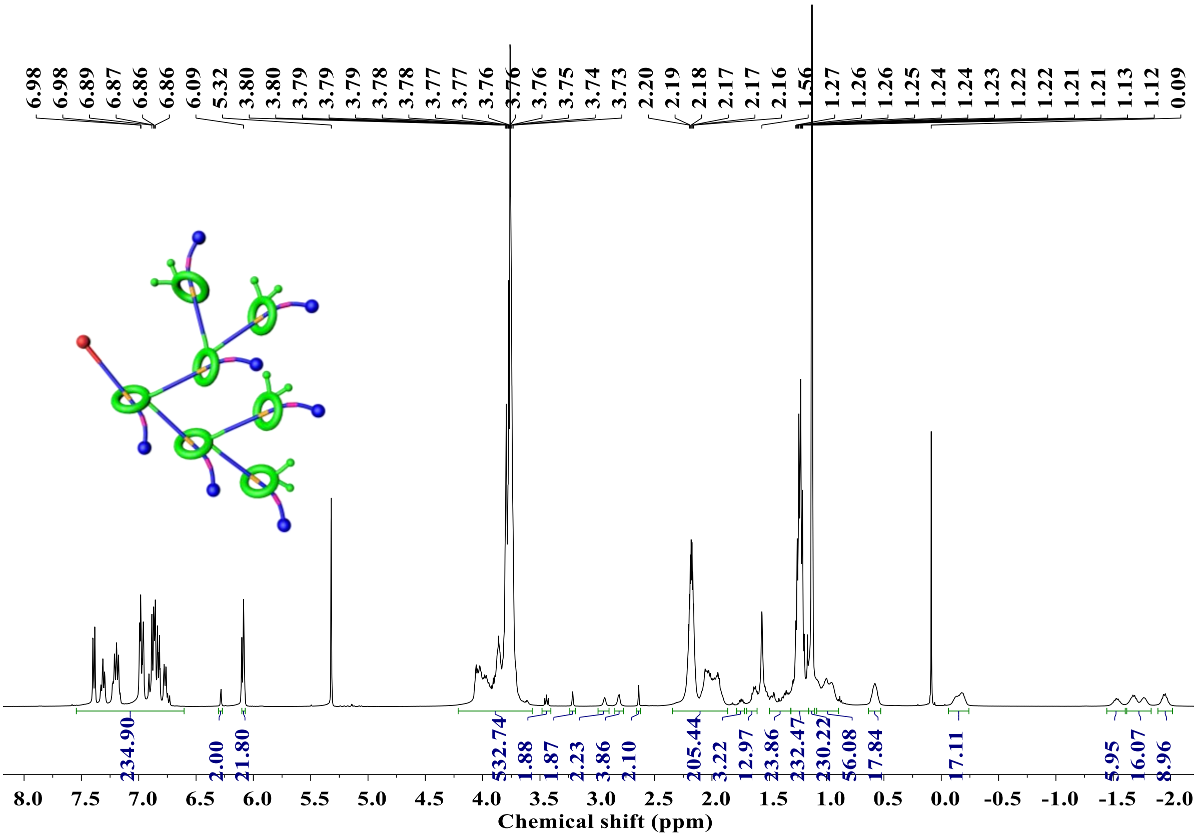

Supplementary Fig. 28 ^{1}H NMR spectrum (CD_2Cl_2, 298 K, 500 MHz) of macromonomer **MG3**.

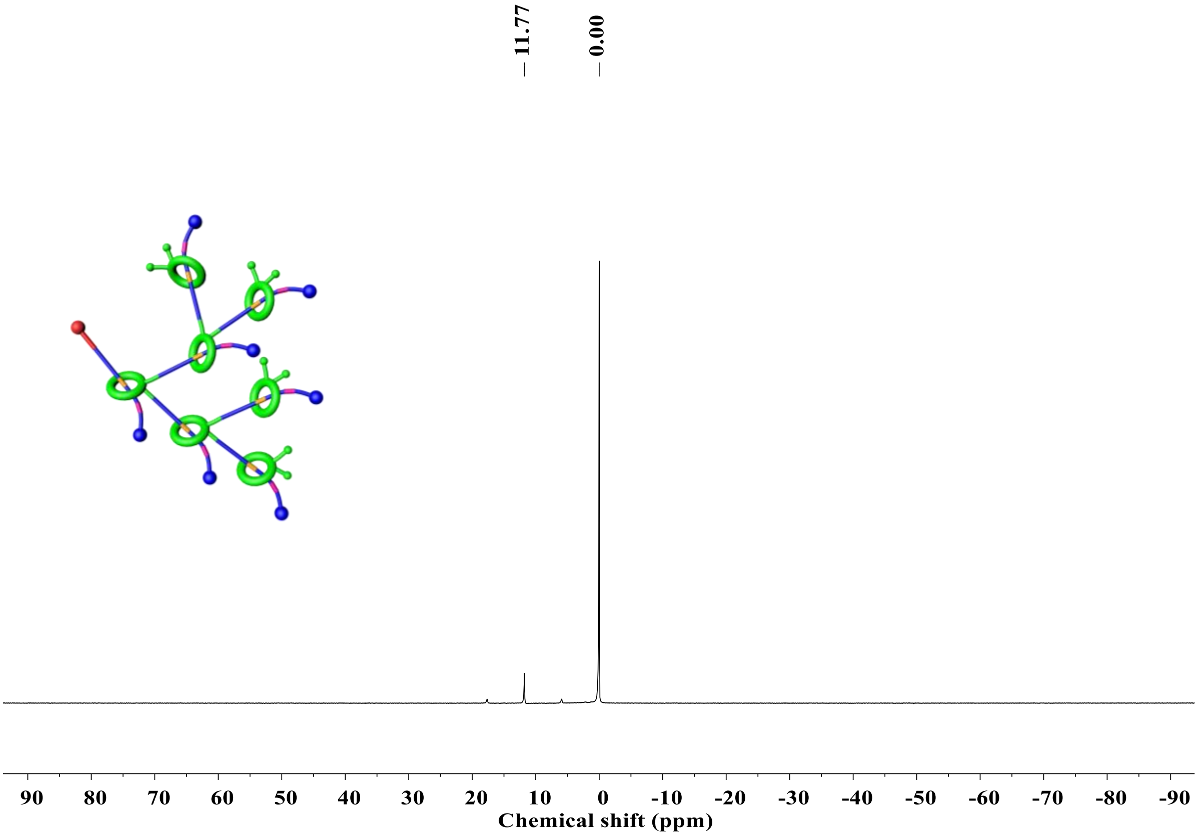

Supplementary Fig. 29 ^{31}P NMR spectrum (CD_2Cl_2, 298 K, 202 MHz) of macromonomer **MG3**.

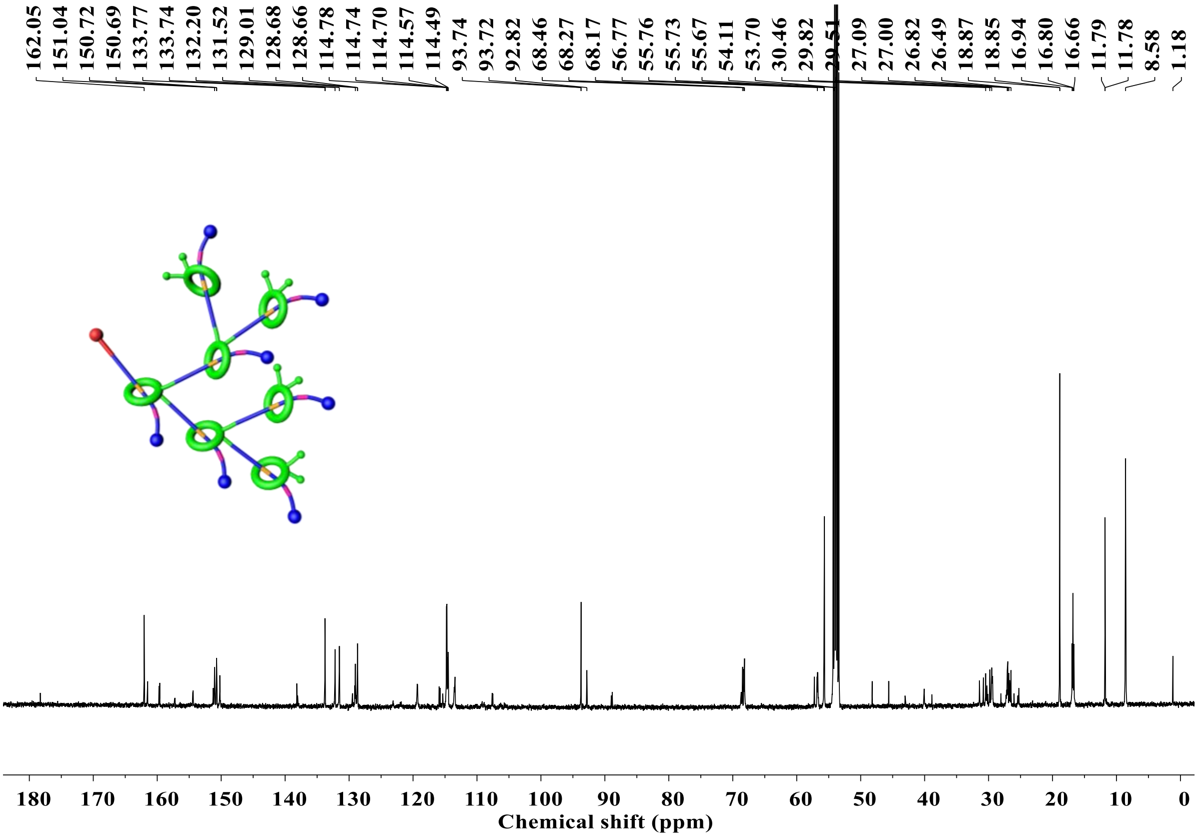

Supplementary Fig. 30 ^{13}C NMR spectrum (CD_2Cl_2, 298 K, 126 MHz) of macromonomer **MG3**.

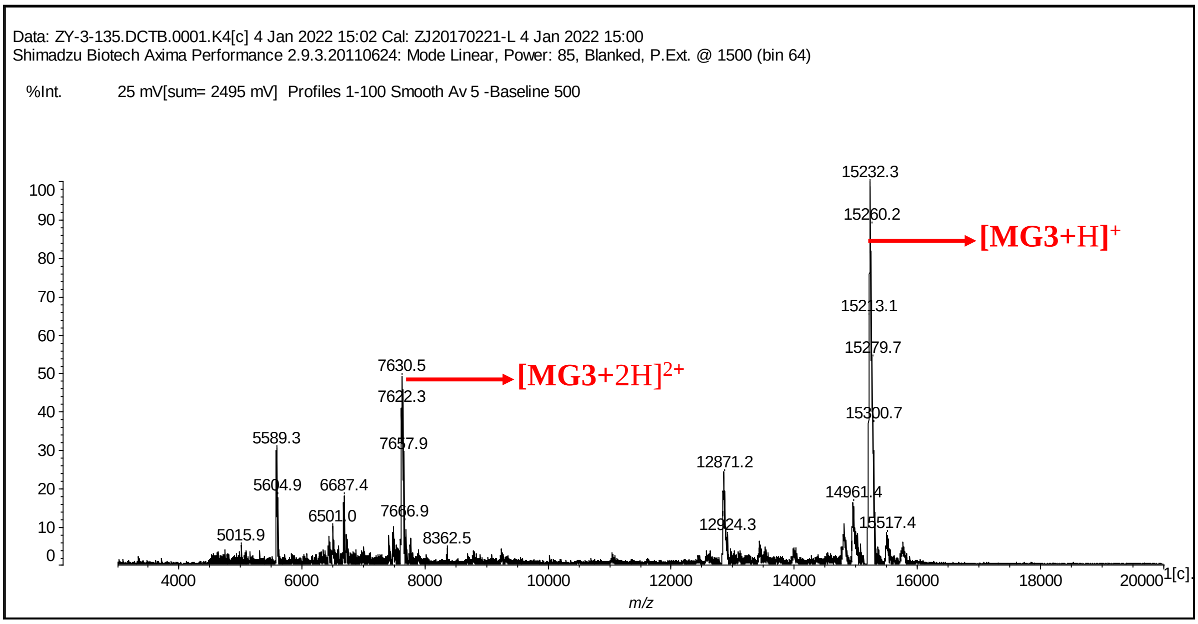

Supplementary Fig. 31 MALDI-TOF-MS spectrum of macromonomer **MG3**.

3. Anion-induced thickness modulation of rotaxane-branched dendrimer macromonomers MGn

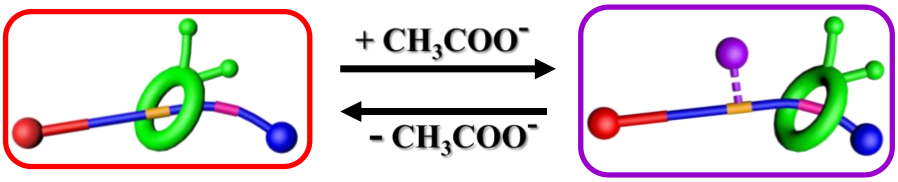

Supplementary Fig. 32 Cartoon representation of anion-induced switching motion of the first-generation rotaxane-branched dendrimer macromonomer **MG1**.

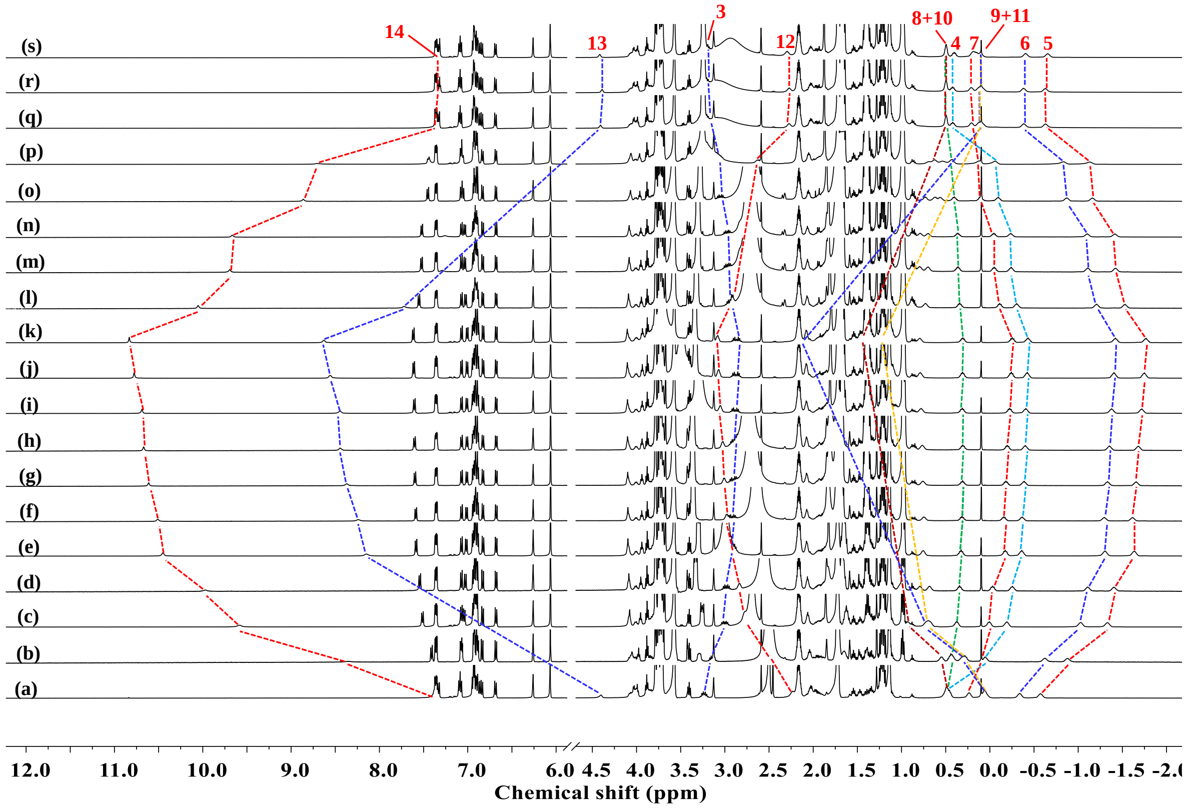

Supplementary Fig. 33 Partial ^{1}H NMR spectra (THF-d_8, 298 K, 500 MHz) of anion-induced switching behavior of the first-generation rotaxane-branched dendrimer macromonomer **MG1**. (a) **MG1**; the mixture of **MG1** and TBAA, for each rotaxane unit: (b) TBAA (0.5 equiv.); (c) TBAA (1.0 equiv.); (d) TBAA (1.5 equiv.); (e) TBAA (2.0 equiv.); (f) TBAA (2.5 equiv.); (g) TBAA (3.0 equiv.); (h) TBAA (3.5 equiv.); (i) TBAA (4.0 equiv.); (j) TBAA (4.5 equiv.); (k) TBAA (5.0 equiv.); and the mixture obtained after adding $NaPF_6$ to the solution in (k), for each rotaxane unit: (l) $NaPF_6$ (1.0 equiv.); (m) $NaPF_6$ (2.0 equiv.); (n) $NaPF_6$ (3.0 equiv.); (o) $NaPF_6$ (4.0 equiv.); (p) $NaPF_6$ (5.0 equiv.); (q) $NaPF_6$ (6.0 equiv.); (r) $NaPF_6$ (8.0 equiv.); (s) $NaPF_6$ (10.0 equiv.).

The optimized structures of one repeat unit of rotaxane-branched DP **PG1** before and after the addition of TBAA have been calculated with the aid of the MOPAC2016 program. As shown in Supplementary Fig. 34, remarkable stretching of the dendron is observed upon the addition of acetate anions as external stimulus. In the initial state, the distance of N1 (-CONCO-)-Si (TIPS) is 44.72 Å. Upon the complexation with acetate anion that triggers the pillar[5]arene macrocycles to move from urea moiety to the alkyl chain station, the distance of N1 (-CONCO-)-Si (TIPS) become 54.15 Å, which indicates the cross-sectional radius (R_{cs}) values of the rotaxane-branched DP PG1 increase after the addition of acetate anions.

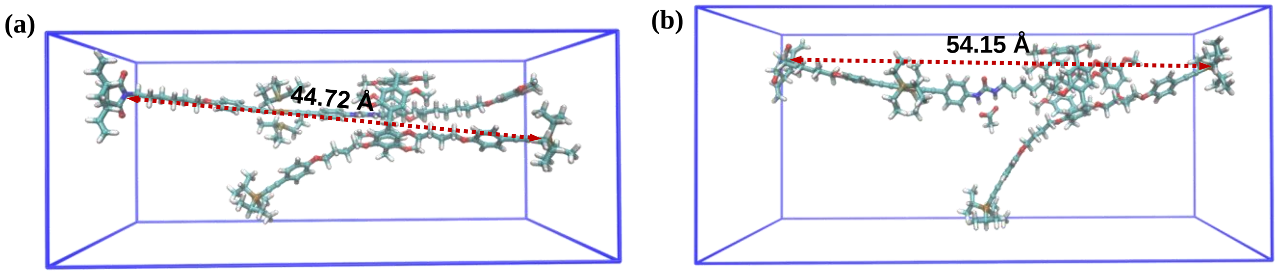

Supplementary Fig. 34 Optimized structures of one repeat unit of rotaxane-branched DP **PG1** before (a) and after (b) the addition of TBAA as stimulus with the aid of the MOPAC2016 program[4,5]. Analytical frequency computations were carried out at the same theoretical level for all stationary points to verify them as intermediate with no imaginary frequency. Structure volume and size variations were analyzed using Multiwfn Software[6]. The visualization of the structures was generated by VMD software[7].

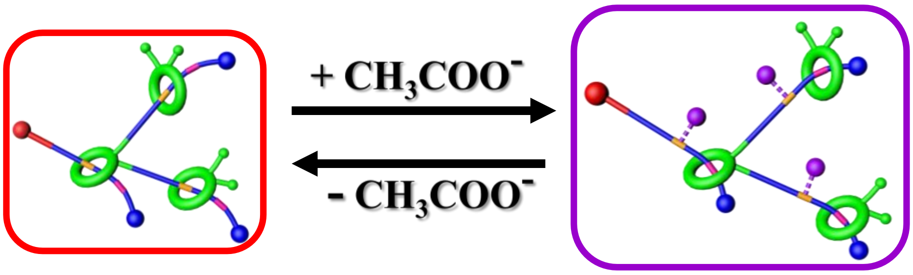

Supplementary Fig. 35 Cartoon representation of anion-induced switching motion of the second-generation rotaxane-branched dendrimer macromonomer **MG2**.

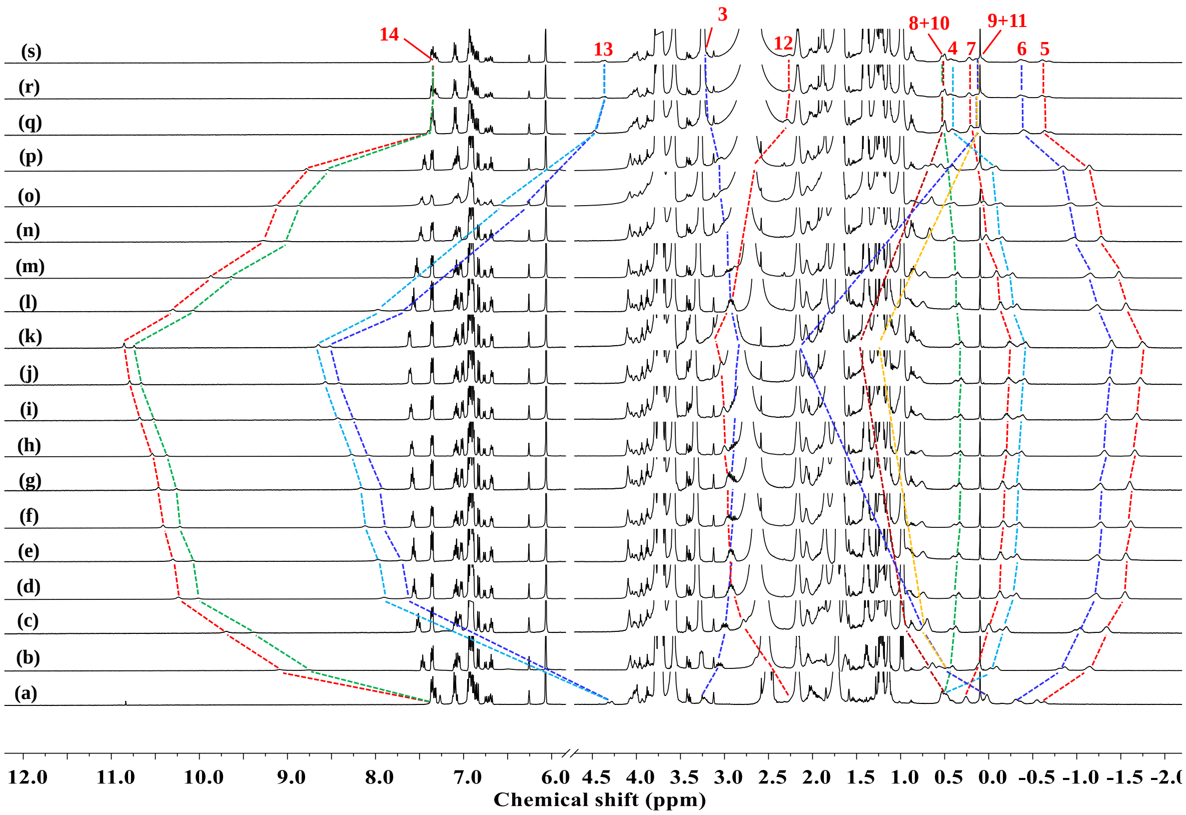

Supplementary Fig. 36 Partial ^{1}H NMR spectra (THF-d_8, 298 K, 500 MHz) of anion-induced switching behavior of the second-generation rotaxane-branched dendrimer macromonomer **MG2**. (a) **MG2**; the mixture of **MG2** and TBAA, for each rotaxane unit: (b) TBAA (0.5 equiv.); (c) TBAA (1.0 equiv.); (d) TBAA (1.5 equiv.); (e) TBAA (2.0 equiv.); (f) TBAA (2.5 equiv.); (g) TBAA (3.0 equiv.); (h) TBAA (3.5 equiv.); (i) TBAA (4.0 equiv.); (j) TBAA (4.5 equiv.); (k) TBAA (5.0 equiv.); and the mixture obtained after adding $NaPF_6$ to the solution in (k), for each rotaxane unit: (l) $NaPF_6$ (1.0 equiv.); (m) $NaPF_6$ (2.0 equiv.); (n) $NaPF_6$ (3.0 equiv.); (o) $NaPF_6$ (4.0 equiv.); (p) $NaPF_6$ (5.0 equiv.); (q) $NaPF_6$ (6.0 equiv.); (r) $NaPF_6$ (8.0 equiv.); (s) $NaPF_6$ (10.0 equiv.).

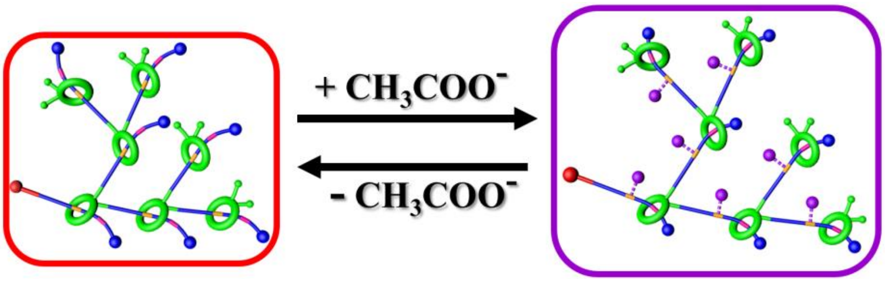

Supplementary Fig. 37 Cartoon representation of anion-induced switching motion of the third-generation rotaxane-branched dendrimer macromonomer **MG3**.

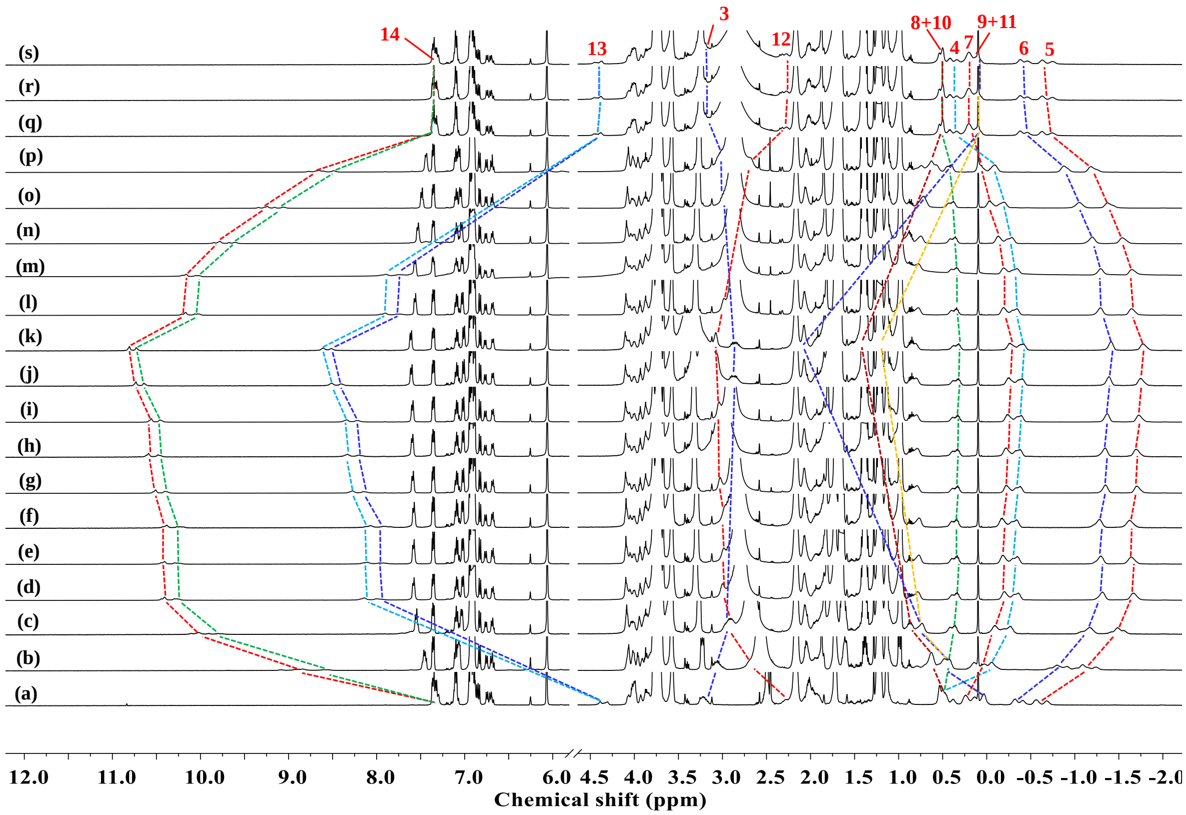

Supplementary Fig. 38 Partial ^{1}H NMR spectra (THF-d_8, 298 K, 500 MHz) of anion-induced switching behavior of the third-generation rotaxane-branched dendrimer macromonomer **MG3**. (a) **MG3**; the mixture of **MG3** and TBAA, for each rotaxane unit: (b) TBAA (0.5 equiv.); (c) TBAA (1.0 equiv.); (d) TBAA (1.5 equiv.); (e) TBAA (2.0 equiv.); (f) TBAA (2.5 equiv.); (g) TBAA (3.0 equiv.); (h) TBAA (3.5 equiv.); (i) TBAA (4.0 equiv.); (j) TBAA (4.5 equiv.); (k) TBAA (5.0 equiv.); and the mixture obtained after adding $NaPF_6$ to the solution in (k), for each rotaxane unit: (l) $NaPF_6$ (1.0 equiv.); (m) $NaPF_6$ (2.0 equiv.); (n) $NaPF_6$ (3.0 equiv.); (o) $NaPF_6$ (4.0 equiv.); (p) $NaPF_6$ (5.0 equiv.); (q) $NaPF_6$ (6.0 equiv.); (r) $NaPF_6$ (8.0 equiv.); (s) $NaPF_6$ (10.0 equiv.).

4. Synthesis and characterizations of rotaxane-branched DPs

General procedure for the synthesis of rotaxane-branched DPs PGn by ring-opening metathesis polymerization: In a nitrogen-filled glovebox, a 4 mL sized screw-cap vial with septum was charged with monomer and a magnetic bar. Anhydrous and degassed THF was added to the vial. The solution of initiator was added at once under vigorous stirring at 40 °C. After 4 h, the reaction was quenched by excess ethyl vinyl ether. The concentrated reaction mixture was precipitated into methanol, and the obtained off-white powder was dried in vacuo.

Procedure for preparation of initiator standard solution: In a nitrogen-filled glovebox, a 1 mL volumetric flask was charged with 4.4 mg of **Ru-III**. The catalyst was then dissolved in a small amount of degassed THF and the resulting solution was made up to 1 mL, to yield a standard solution having a concentration of 5×10^{-3} M.

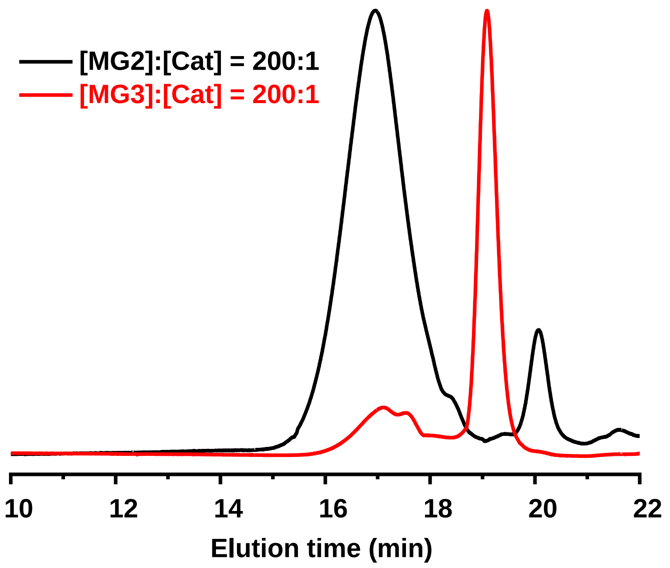

Supplementary Fig. 39 GPC traces of the polymerization systems for the synthesis of **PG2** and **PG3** with the feed ratio of monomer to initiator ([M]/[I]) of 200:1.

Supplementary Table 1 Polymerizations of macromonomers[a]

Entry	[**MG1**]:[**MG2**]:[**MG3**] : [I][b]	Time (h)	Yield (%)	M_w[c] (kDa)	$Đ$[c]
1	200:0:0:1	4	92	498.4	1.28
2	0:100:0:1	4	84	402.5	1.21
3	0:0:50:1	4	80	433.4	1.23

[a]Polymerization conditions: T = 40 °C, I = **Ru-III**, solvent = THF, the initial concentration of **MG1** was 0.1 mol mL^{-1}、**MG2** was 0.04 mol mL^{-1} and **MG3** was 0.01mol L^{-1}, respectively. [b] The feed ratios of macromonomer to catalyst. [c]Molecular weight (M_w) and polydispersity index ($Đ$) of the single

polymer chain determined by GPC using THF as eluent. The weight-average molar mass (M_w) and polydispersity index (*Đ*) were determined by GPC-MALLS.

Synthesis of the first-generation rotaxane-branched DP PG1: In a nitrogen-filled glovebox, a 4 mL sized screw-cap vial with septum was charged with **MG1** (263 mg, 0.1 mmol) and a magnetic bar. The monomer was dissolved in 0.9 mL of dry THF and 100 μL of the catalyst solution was rapidly injected under vigorous stirring at 40 °C for 4 h, the reaction was quenched by excess ethyl vinyl ether. The concentrated reaction mixture was precipitated into methanol, and the obtained off-white powder was dried in vacuo (243 mg, 92%). ^{1}H NMR (400 MHz, CD_2Cl_2, 298 K): δ 7.41-7.36 (d), 7.32-7.28 (d), 7.22-7.14 (t), 6.99-6.96 (d), 6.89-6.79 (m), 6.76-6.72 (d), 6.07 (s), 5.85-5.70 (br), 5.50-5.48 (br), 4.08-3.70 (m), 3.49-3.41 (bs), 3.10-2.90 (br), 2.85-2.76 (s), 2.75-2.65 (br), 2.22-2.12 (m), 2.10-1.89(m), 1.79-1.71 (s), 1.67-1.60 (bs), 1.50-1.46 (m), 1.40-1.33 (m), 1.26-1.20 (m), 1.13 (s), 1.05-0.90 (m), 0.57 (bs), -0.18 (bs), -1.67(bs). -1.94 (bs). ^{31}P NMR (162 MHz, CD_2Cl_2): δ 11.42. GPC: M_w = 498.4 kDa, *Đ* = 1.28.

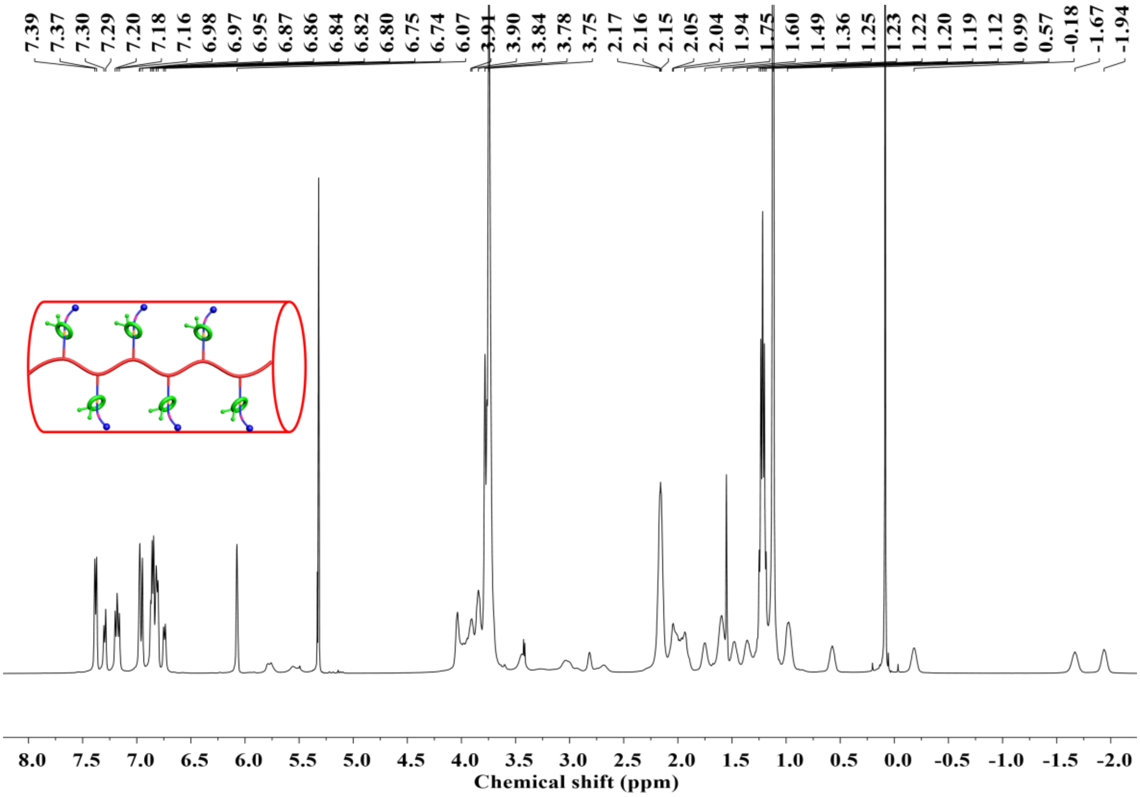

Supplementary Fig. 40 ^{1}H NMR spectrum (CD_2Cl_2, 298 K, 400 MHz) of rotaxane-branched DP **PG1**.

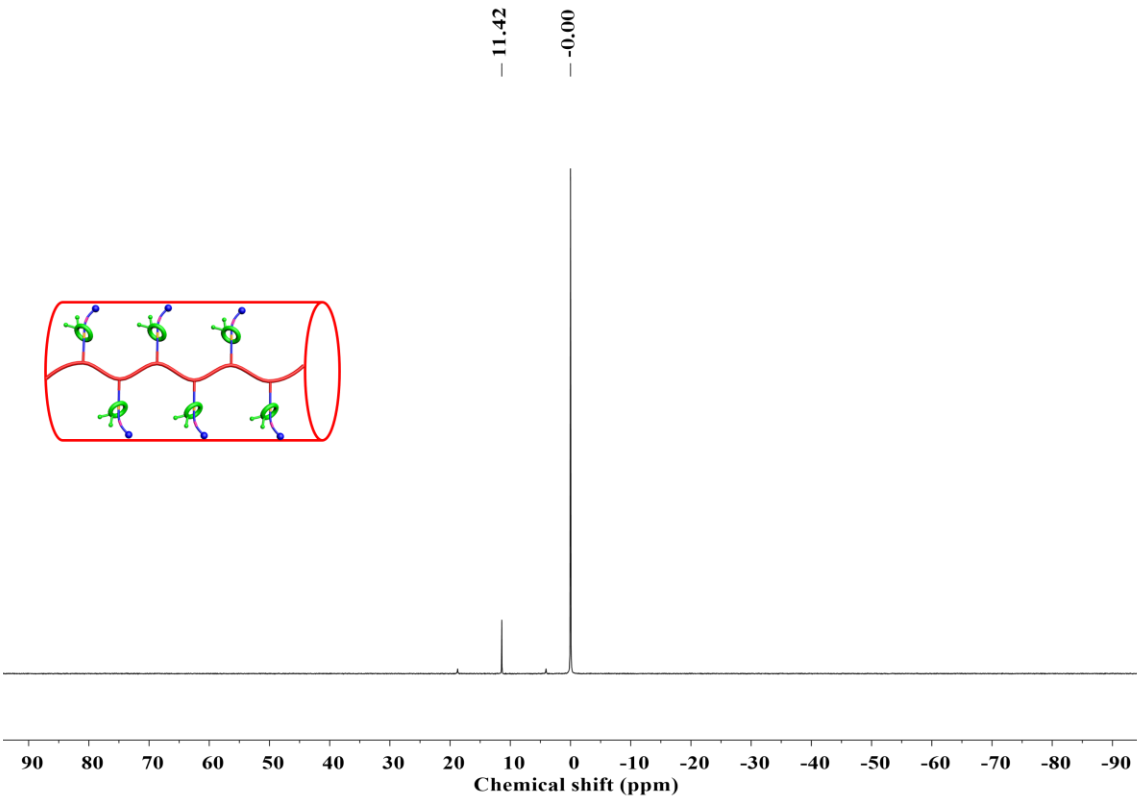

Supplementary Fig. 41 ^{31}P NMR spectrum (CD_2Cl_2, 298 K, 162 MHz) of rotaxane-branched DP **PG1**.

Synthesis of the second-generation rotaxane-branched DP PG2: In a nitrogen-filled glovebox, a 4 mL sized screw-cap vial with septum was charged with **MG2** (274 mg, 0.04 mmol) and a magnetic bar. The monomer was dissolved in 0.96 mL of dry THF and 40 μL of the catalyst solution was rapidly injected under vigorous stirring at 40 ºC for 4 h, the reaction was quenched by excess ethyl vinyl ether. The concentrated reaction mixture was precipitated into methanol, and the obtained off-white powder was dried in vacuo (230 mg, 84%). ^{1}H NMR (400 MHz, CD_2Cl_2, 298 K): δ 7.41-7.37 (d), 7.33-7.28 (m), 7.24-7.15 (m), 7.01-6.93 (m), 6.92-6.80 (m), 6.79-6.72 (m), 6.08 (m), 5.80-5.70 (br), 5.59-5.45 (br), 4.09-3.66 (m), 3.44 (s), 3.10-2.90 (br), 2.85-2.76 (s), 2.75-2.60 (br), 2.25-2.12 (m), 2.08-1.90 (m), 1.75 (s), 1.65-1.55 (m), 1.52-1.54 (m), 1.32-1.19 (m), 1.12 (s), 1.05-0.92 (m), 0.63-0.51 (br), -0.17 (br), -1.52 (bs), -1.60--1.80 (m), -1.92--2.00 (m). ^{31}P NMR (162 MHz, CD_2Cl_2): δ 11.41, 11.37. GPC: M_w = 402.5 kDa, $Đ$ = 1.21.

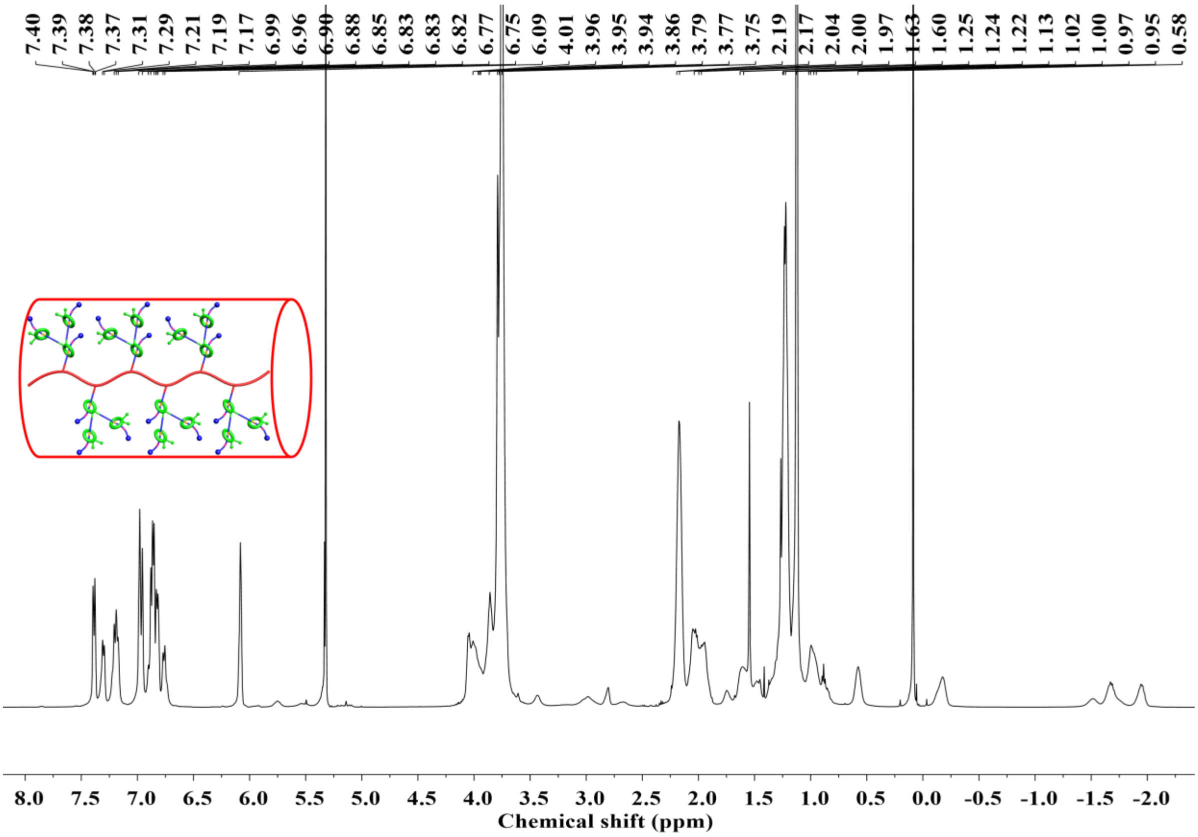

Supplementary Fig. 42 ^{1}H NMR spectrum (CD_2Cl_2, 298 K, 400 MHz) of rotaxane-branched DP **PG2**.

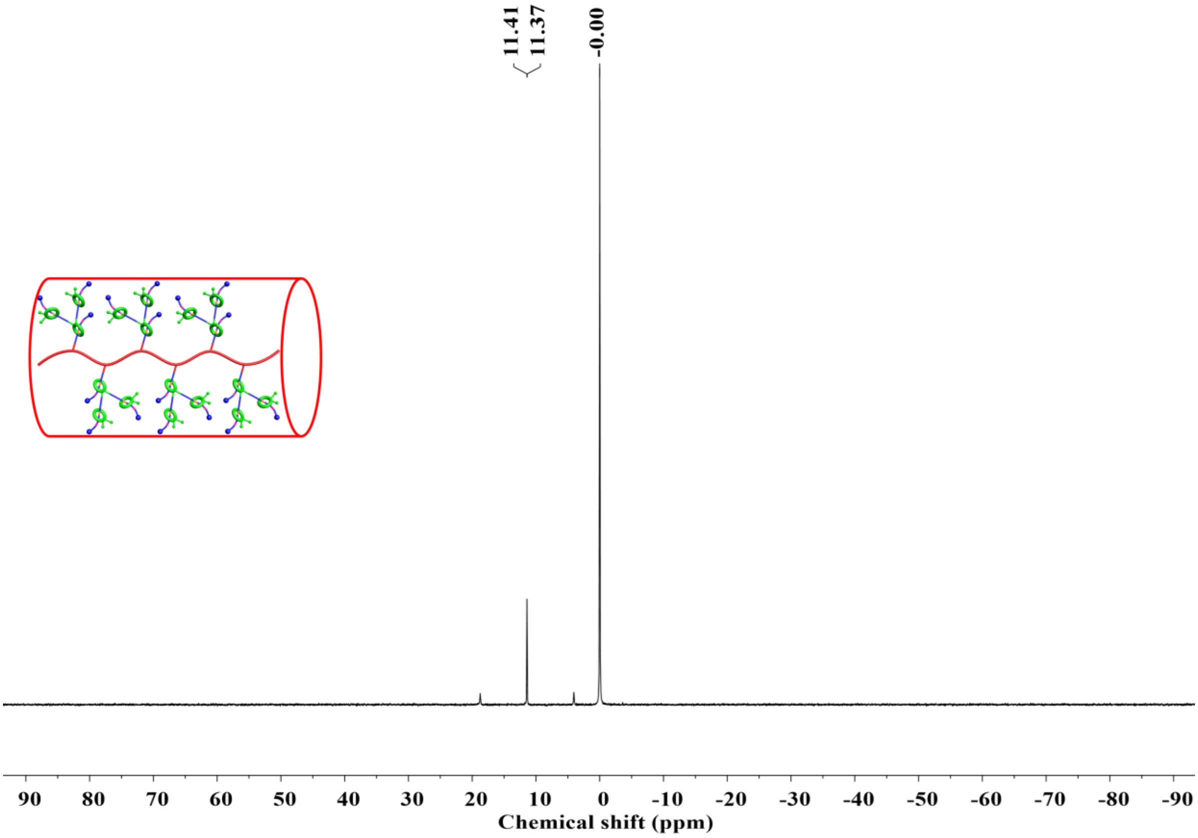

Supplementary Fig. 43 ^{31}P NMR spectrum (CD_2Cl_2, 298 K, 162 MHz) of rotaxane-branched DP **PG2**.

Synthesis of the third-generation rotaxane-branched DP PG3: In a nitrogen-filled glovebox, a 4 mL sized screw-cap vial with septum was charged with **MG3** (152 mg, 0.01mmol) and a magnetic bar. The monomer was dissolved in 0.96 mL of dry THF and 40 μL of the catalyst solution was rapidly injected under vigorous stirring at 40 °C for 4 h, the reaction was quenched by excess ethyl vinyl ether. The concentrated reaction mixture was precipitated into methanol, and the obtained off-white powder was dried in vacuo (120 mg, 80%). ^{1}H NMR (400 MHz, CD_2Cl_2, 298 K): δ 7.41-7.36 (bd), 7.33-7.27 (m), 7.23-7.14 (m), 7.00-6.93 (bd), 6.91-6.79 (m), 6.78-6.71 (m), 6.07 (s), 5.80-5.68 (br), 5.59-5.45 (br), 4.10-3.60 (m), 3.48-3.36 (m), 3.10-2.90 (br), 2.90-2.78 (s), 2.75-2.60 (br), 2.21-2.11 (s), 2.08-1.87 (m), 1.74 (s), 1.50-1.31(m), 1.30-1.18 (m), 1.12 (s), 1.04-0.81 (br), 0.62-0.49 (br), -0.19 (bs), -1.52 (bs), -1.62--1.83 (m), -1.88--2.04 (m). ^{31}P NMR (162 MHz, CD_2Cl_2): δ 11.41, 11.38. GPC: M_w = 433.4 kDa, $Đ$ = 1.23.

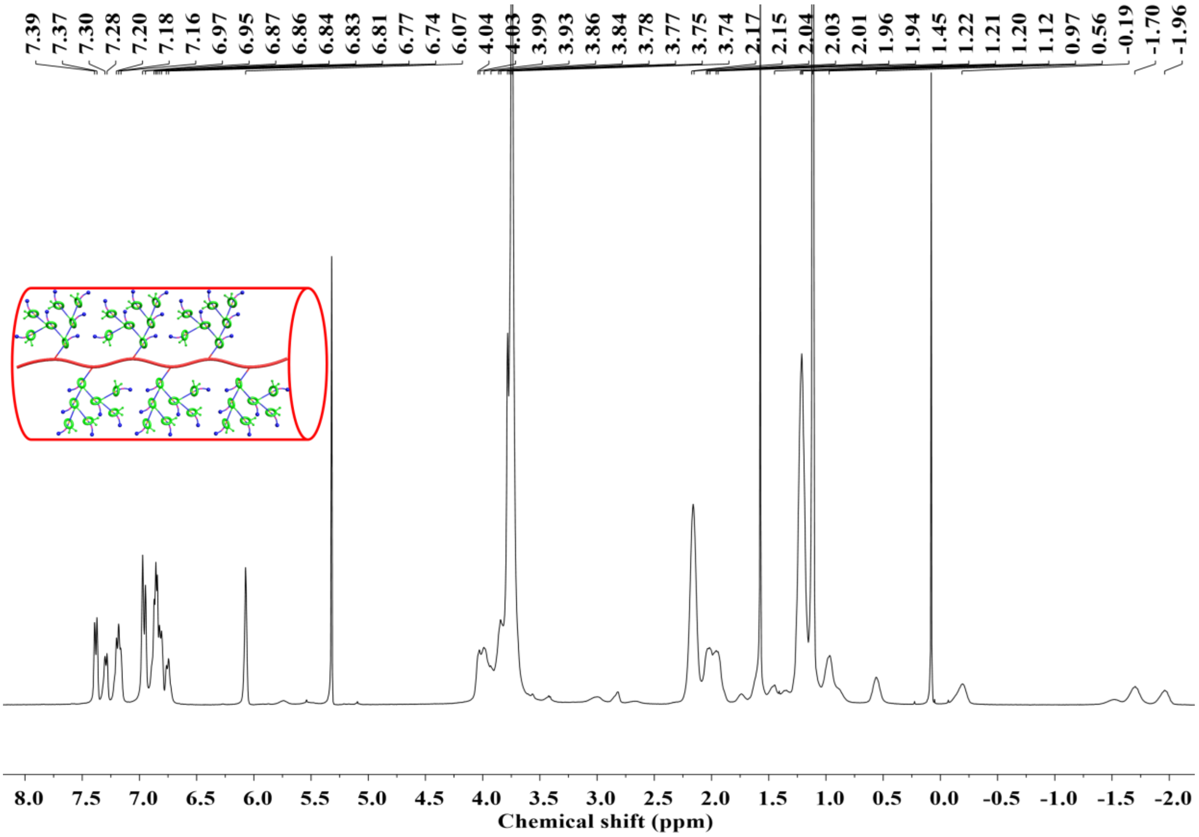

Supplementary Fig. 44 ^{1}H NMR spectrum (CD_2Cl_2, 298 K, 400 MHz) of rotaxane-branched DP **PG3**.

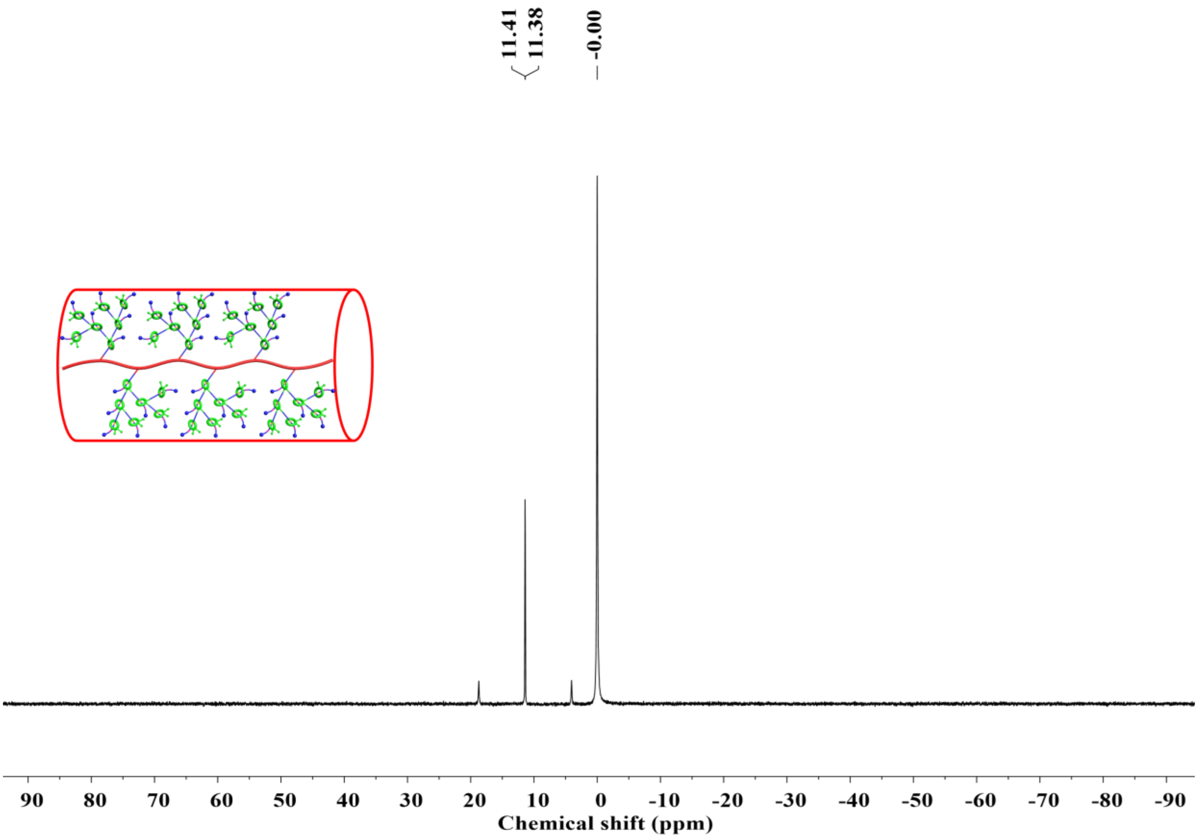

Supplementary Fig. 45 ^{31}P NMR spectrum (CD_2Cl_2, 298 K, 162 MHz) of rotaxane-branched DP **PG3**.

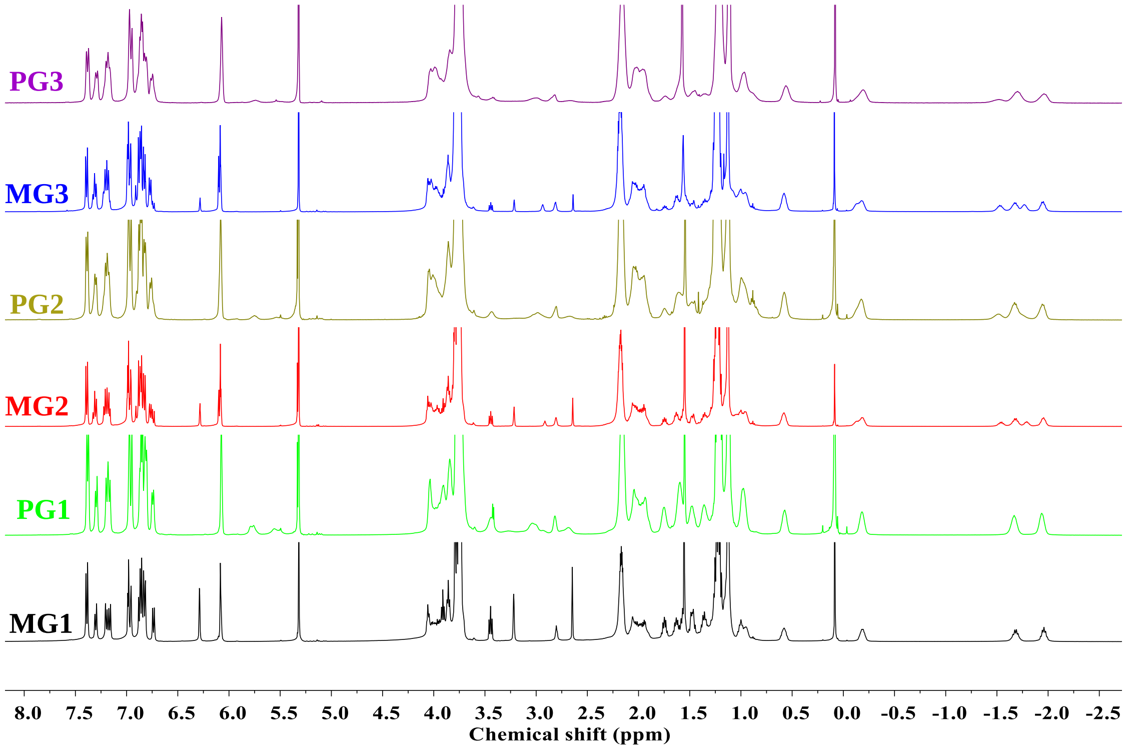

Supplementary Fig. 46 ^{1}H NMR spectra of macromonomers **MG1-MG3** and corresponding rotaxane-branched DPs **PG1-PG3**.

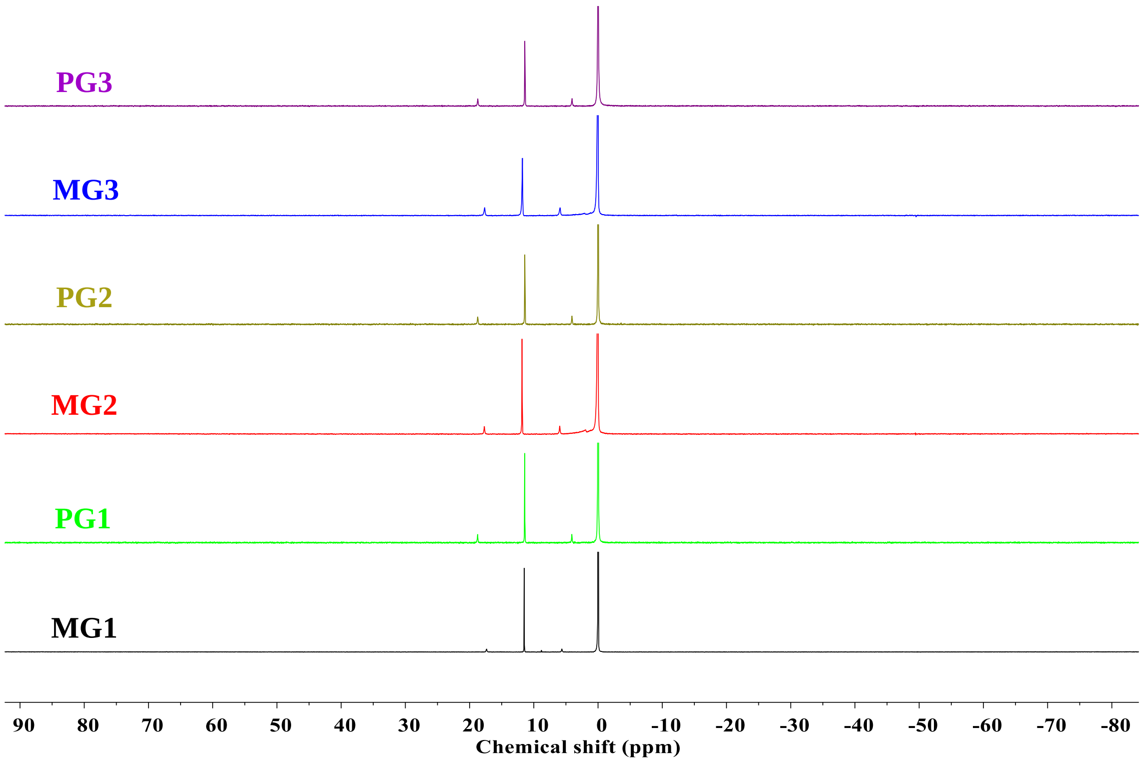

Supplementary Fig. 47 ^{31}P NMR spectra of macromonomers **MG1-MG3** and corresponding rotaxane-branched DPs **PG1-PG3**.

5. Small angle neutron scattering (SANS) experiments

The small angle neutron scattering (SANS) experiment were carried out at the SANS beamline at China Spallation neutron source. A wavelength ranges from 1.2 to 9.5Å, corresponds to q-range 0.005-0.8Å^{-1}, was employed during the measurements. Samples prepared in deuterated tetrahydrofuran (THF-d_8) were loaded in quartz Banjo cells with 2 mm light path. The final scattering profile of the samples have been corrected for the sample transmission, scattering from solvent and quartz cell, instrument background and detector efficiency, and were calibrated to absolute scale with a secondary standard sample provided by the beamline. The indirect Fourier transformation of the data was performed using the pair distance distribution function tool provided in Irena package[8] based on Igor, while the model dependent data fitting was performed using SasView[9].

The SANS data were fitted with unified function used to fit scattering curves from a fractal object with several structural levels following the scattering function:

$$I(q) = \sum_i \left(G_i\, e^{-\frac{R_{g,i}^2 q^2}{3}} + B_i\, e^{-\frac{R_{g,i-1}^2 q^2}{3}} (q_i^*)^{-d_{f,i}} \right) \qquad [1]$$

where $q_i^* = \frac{q}{\mathrm{erf}(\frac{q\,1.06\,R_{g,i}}{\sqrt{3}})^3}$, erf(x) means error function. Index i=1, 2,… correspond to different structure levels, i=1 is defined for the smallest structural level. $R_{g,i}$: Radius of gyration correspond to structural level *I*. $d_{f,i}$: Fractal dimension correspond to structural level *I*. G_i : Guinier law prefactor. B_i: power law prefactor.

All the scattering profiles consist of two Guinier regions, corresponds to two characteristic length scales. The Guinier regions in the high-q range (q~0.2Å^{-1}) of the three samples overlaps, indicating the same scattering origin of this feature, which is the single rotaxane building block. The scattering profiles of the corresponding region were fitted with the unified model[10]. The radius of gyration (R_g) obtained from the data sets agrees well with the one obtained from the SANS data of theoretically evaluated R_g agrees well. The scattering curves in the lower q-range corresponds to the overall structure of the rotaxane-branched dendronized polymers. Based on the shape of the PDDFs of the three samples, specific rigid body models were allocated for each sample. The scattering profiles of **PG1** and **PG2** were fitted with rigid cylinder model while **PG3** was fitted with sphere model. The radius of the sphere and that of the cross-section of the cylinder were assumed to have lognormal distribution.

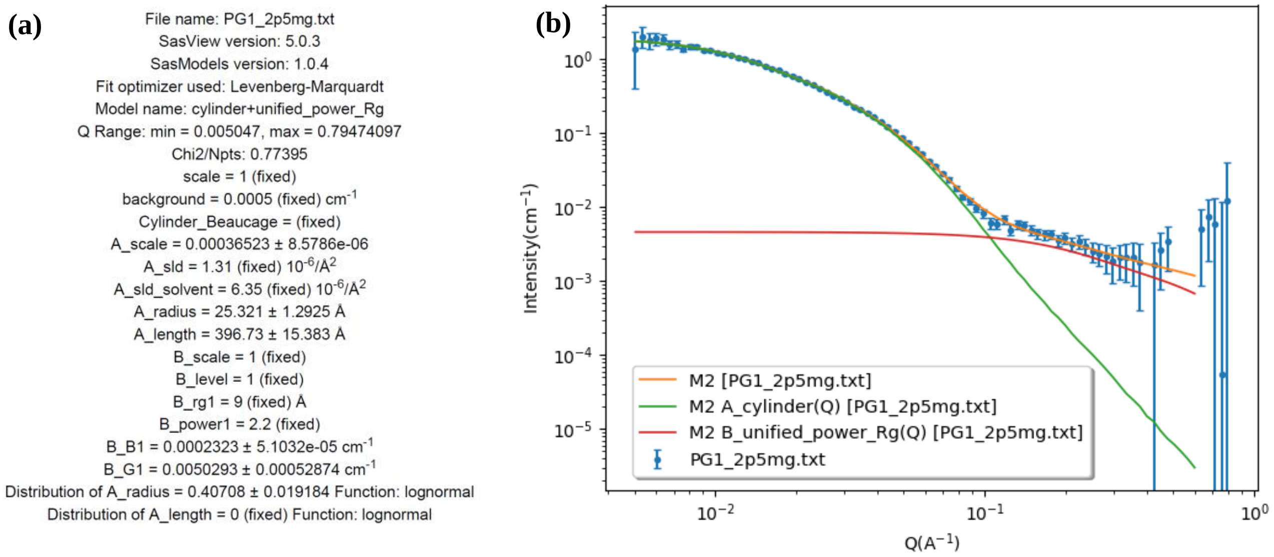

Supplementary Fig. 48 Fitting results of rotaxane-branched DP **PG1**. (a) fitting data. (b) fitting curve.

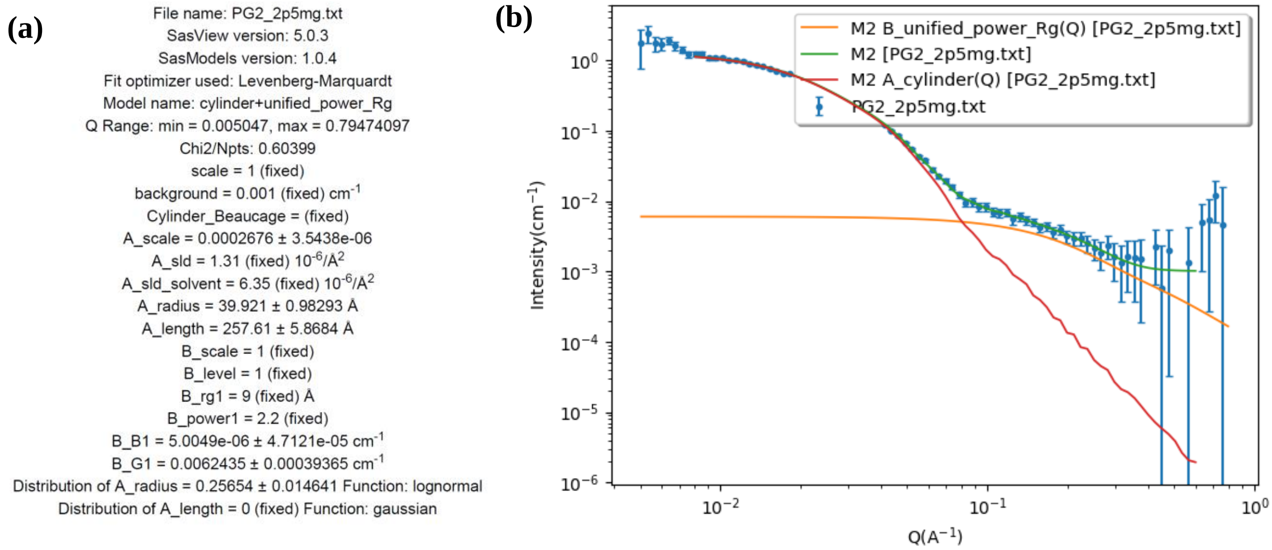

Supplementary Fig. 49 Fitting results of rotaxane-branched DP **PG2**. (a) fitting data. (b) fitting curve.

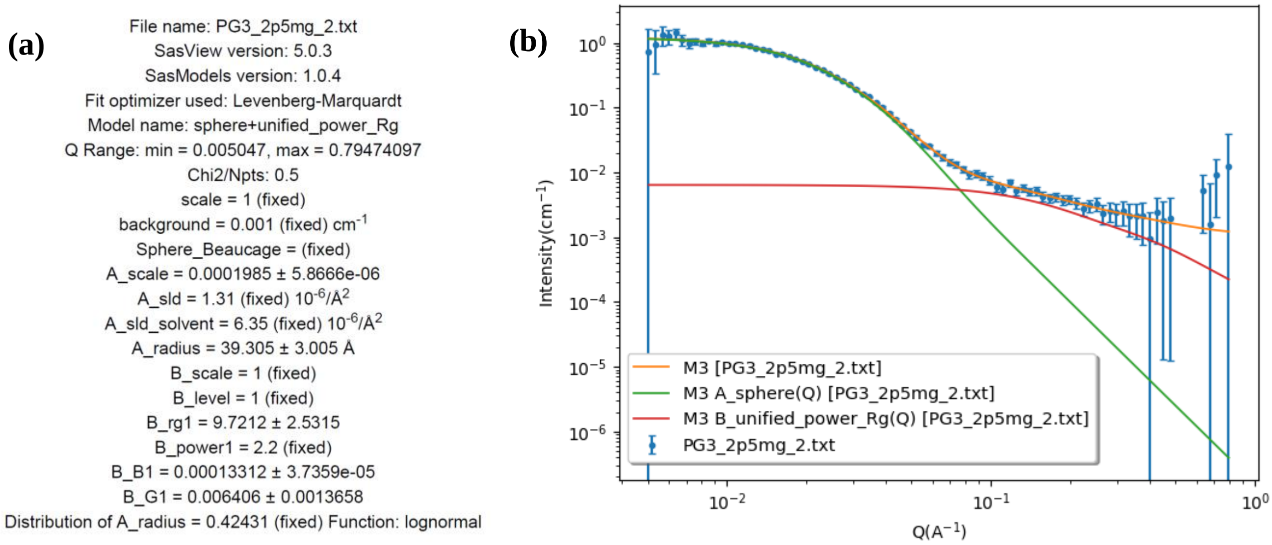

Supplementary Fig. 50 Fitting results of rotaxane-branched DP **PG3**. (a) fitting data. (b) fitting curve.

6. AFM images of rotaxane-branched DPs

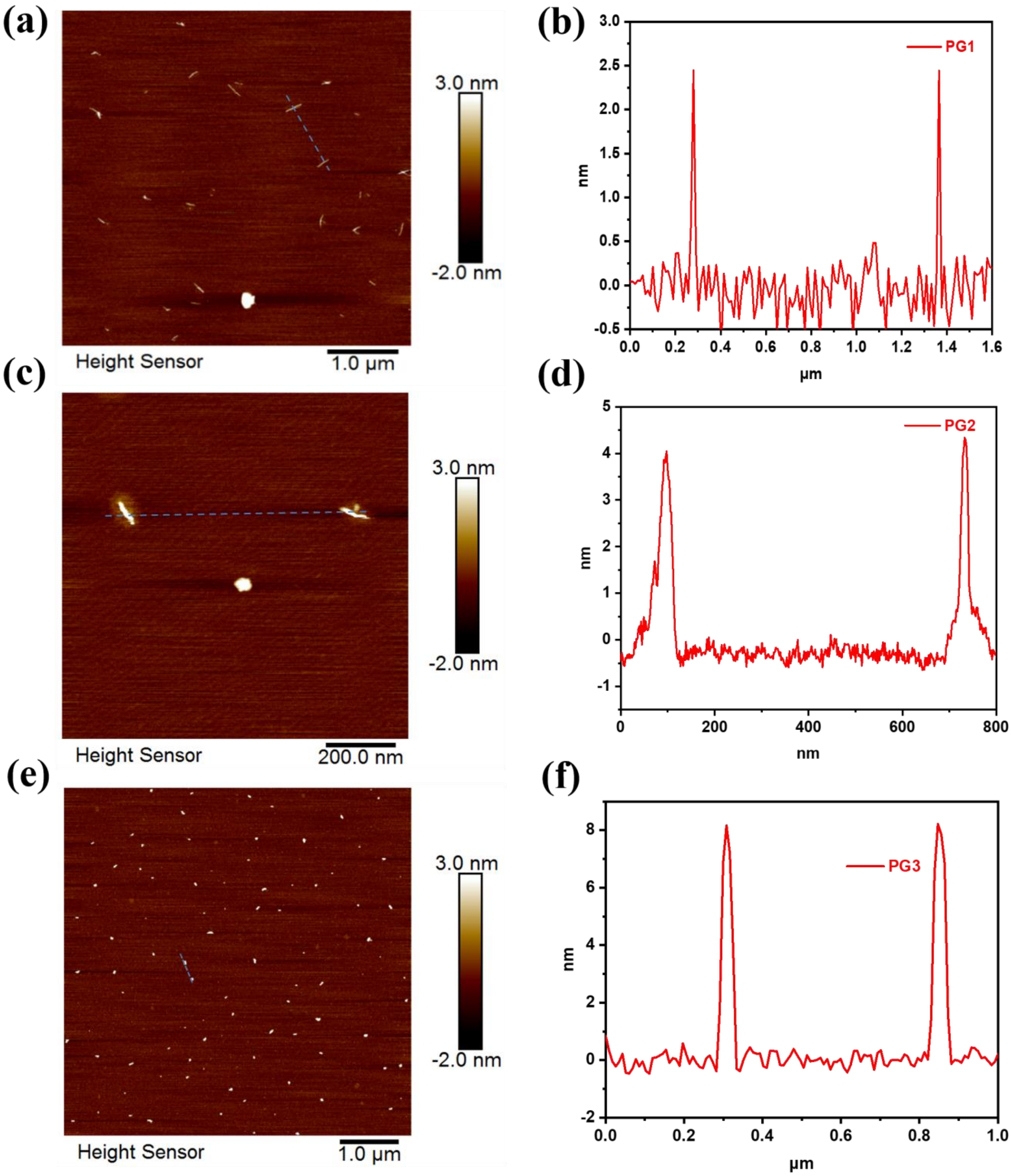

Supplementary Fig. 51 AFM height images of rotaxane-branched DPs on mica surface. (a) **PG1**; (b) the height range of **PG1** is 2.40 ± 0.15 nm; (c) **PG2**; (d) the height range of **PG2** is 4.00 ± 0.10 nm; (e) **PG3**; (f) the height range of **PG3** is 8.00 ± 0.10 nm.

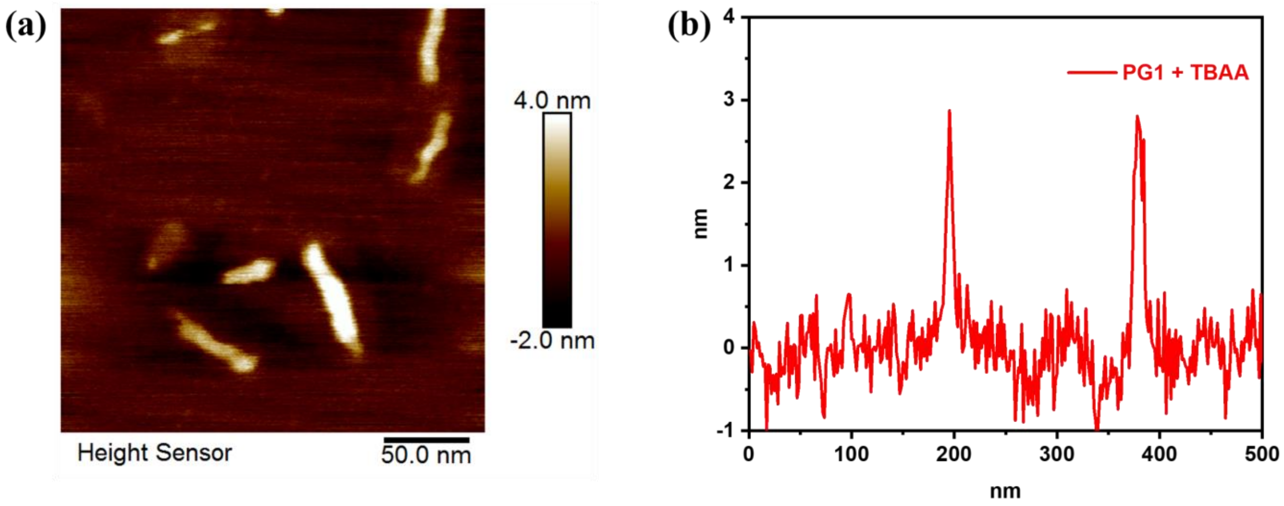

Supplementary Fig. 52 AFM images of rotaxane-branched DPs on mica surface. (a) **PG1** with the addition of TBAA (5eq. for each urea unit); (b) the height range of **PG1** + TBAA is 2.87 ± 0.15 nm.

7. Anion-induced thickness modulation of rotaxane-branched DPs

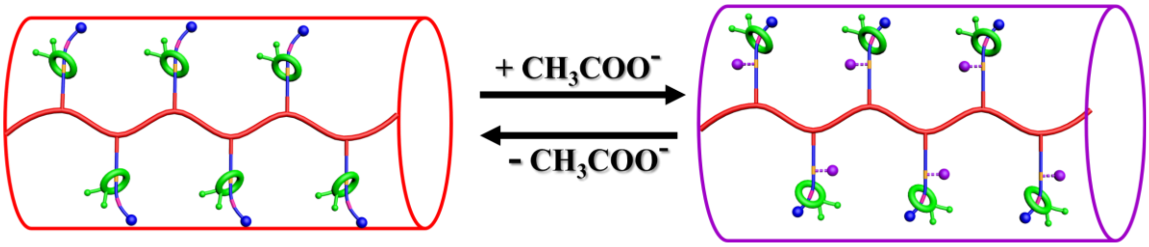

Supplementary Fig. 53 Cartoon representation of anion-induced switching motion of rotaxane-branched DP **PG1**.

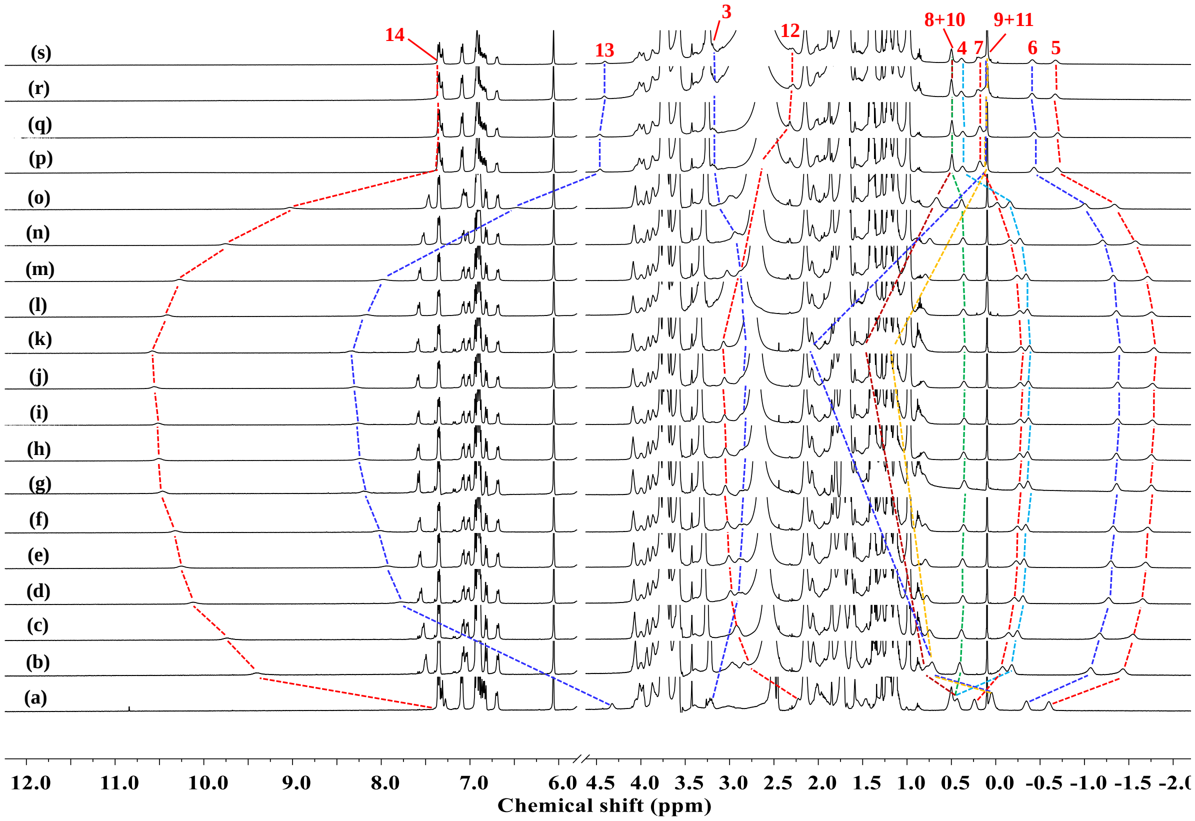

Supplementary Fig. 54 Partial ^1H NMR spectra (THF-d_8, 298 K, 500 MHz) of anion-induced switching behavior of rotaxane-branched DP **PG1**. (a) **PG1**; the mixture of **PG1** and TBAA, for each rotaxane unit: (b) TBAA (0.5 equiv.); (c) TBAA (1.0 equiv.); (d) TBAA (1.5 equiv.); (e) TBAA (2.0 equiv.); (f) TBAA (2.5 equiv.); (g) TBAA (3.0 equiv.); (h) TBAA (3.5 equiv.); (i) TBAA (4.0 equiv.); (j) TBAA (4.5 equiv.); (k) TBAA (5.0 equiv.); and the mixture obtained after adding $NaPF_6$ to the solution in (k), for each rotaxane unit: (l) $NaPF_6$ (1.0 equiv.); (m) $NaPF_6$ (2.0 equiv.); (n) $NaPF_6$ (3.0 equiv.); (o) $NaPF_6$ (4.0 equiv.); (p) $NaPF_6$ (5.0 equiv.); (q) $NaPF_6$ (6.0 equiv.); (r) $NaPF_6$ (8.0 equiv.); (s) $NaPF_6$ (10.0 equiv.).

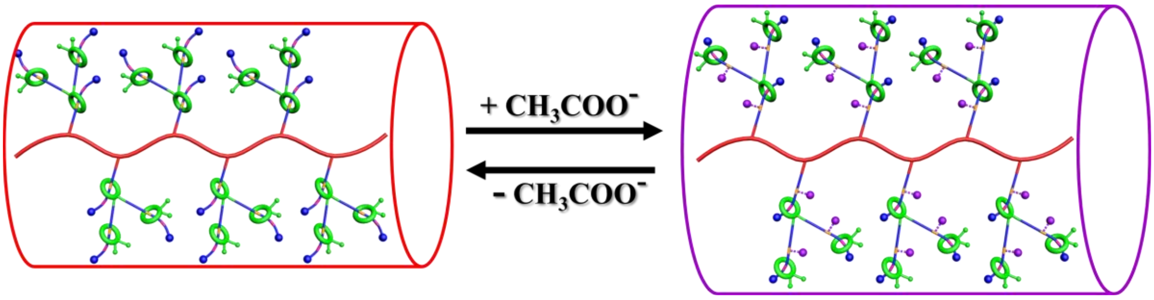

Supplementary Fig. 55 Cartoon representation of anion-induced switching motion of the rotaxane-branched DP **PG2**.

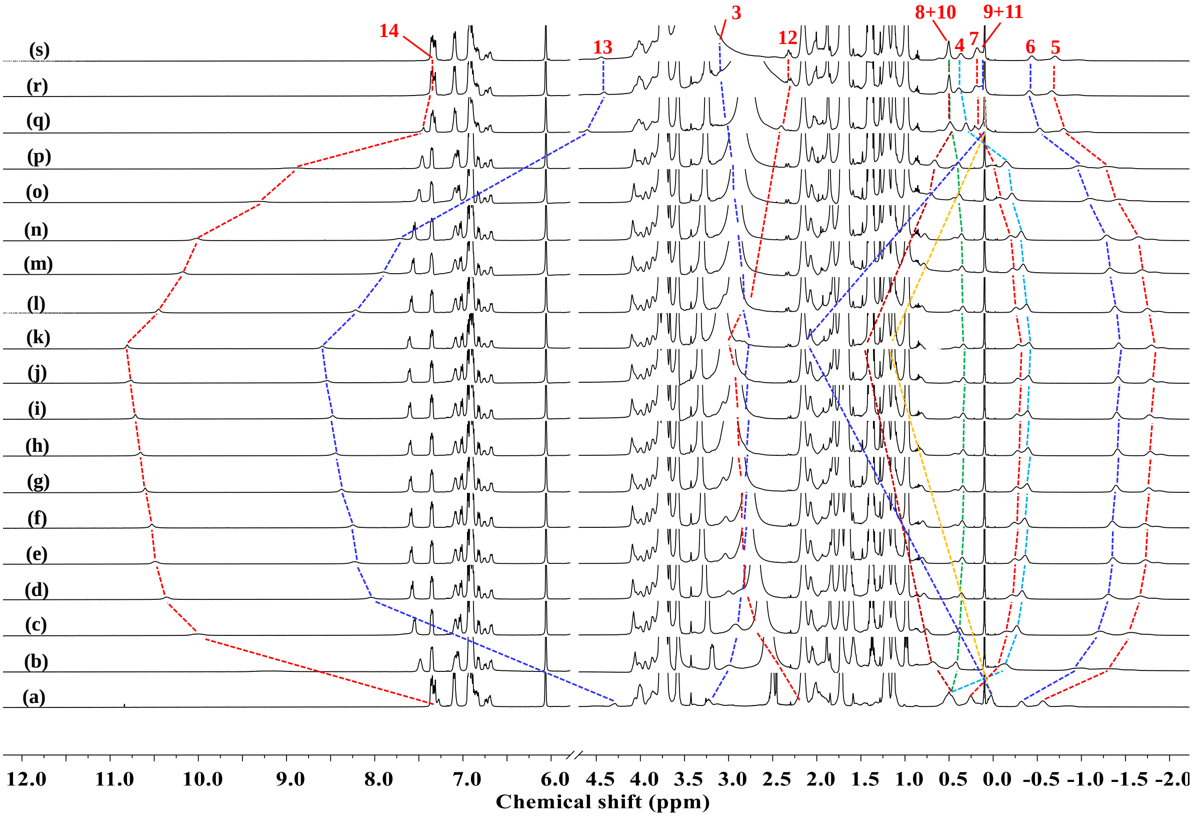

Supplementary Fig. 56 Partial ^{1}H NMR spectra (THF-d_8, 298 K, 500 MHz) of anion-induced switching behavior of rotaxane-branched DP **PG2**. (a) **PG2**; the mixture of **PG2** and TBAA, for each rotaxane unit: (b) TBAA (0.5 equiv.); (c) TBAA (1.0 equiv.); (d) TBAA (1.5 equiv.); (e) TBAA (2.0 equiv.); (f) TBAA (2.5 equiv.); (g) TBAA (3.0 equiv.); (h) TBAA (3.5 equiv.); (i) TBAA (4.0 equiv.); (j) TBAA (4.5 equiv.); (k) TBAA (5.0 equiv.); and the mixture obtained after adding $NaPF_6$ to the solution in (k), for each rotaxane unit: (l) $NaPF_6$ (1.0 equiv.); (m) $NaPF_6$ (2.0 equiv.); (n) $NaPF_6$ (3.0 equiv.); (o) $NaPF_6$ (4.0 equiv.); (p) $NaPF_6$ (5.0 equiv.); (q) $NaPF_6$ (6.0 equiv.); (r) $NaPF_6$ (8.0 equiv.); (s) $NaPF_6$ (10.0 equiv.).

8. Tunable thermal properties of rotaxane-branched DPs

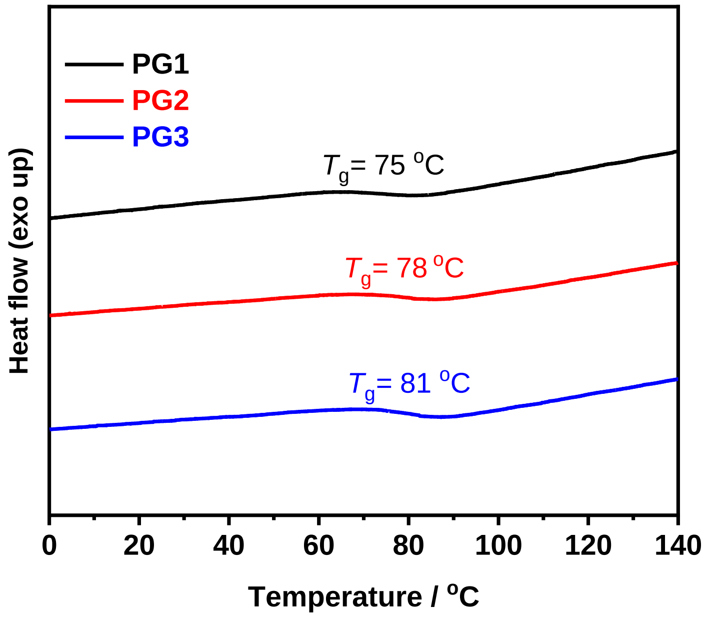

Supplementary Fig. 57 DSC curves of rotaxane-branched DPs **PGn** in the second heating process.

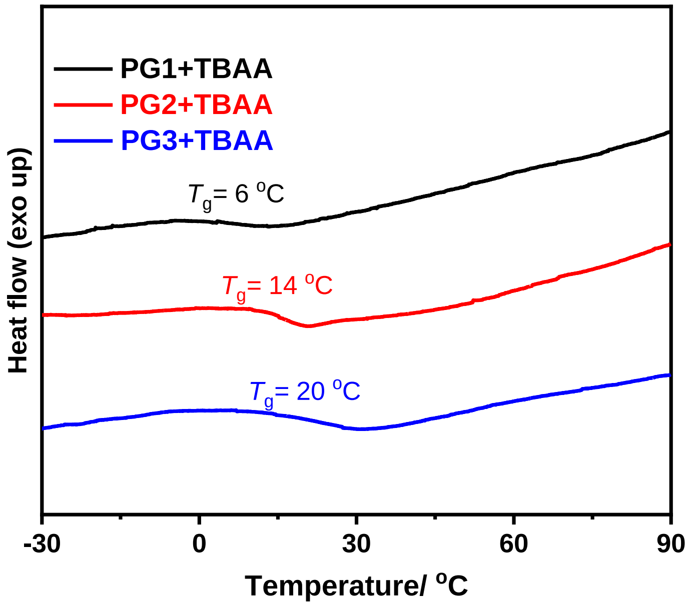

Supplementary Fig. 58 DSC curves of **PGn** + TBAA in the second heating process.

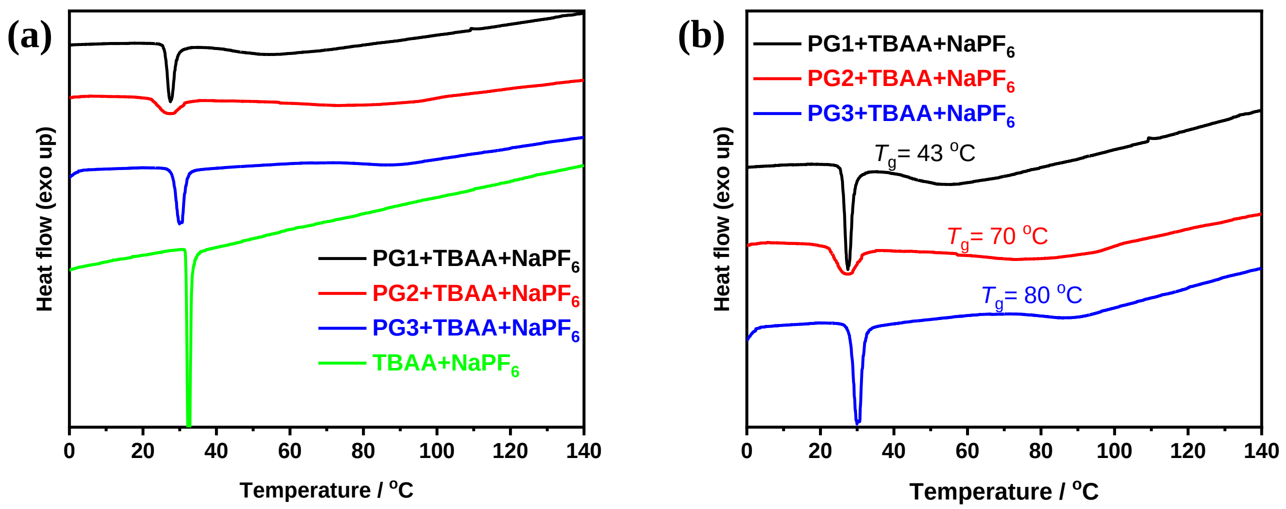

Supplementary Fig. 59 (a) DSC curves of **PGn**+TBAA+$NaPF_6$ and TBAA+$NaPF_6$ in the second heating process. (b) DSC curves of **PGn**+TBAA+$NaPF_6$ and in the second heating process.

9. Tunable rheological properties of rotaxane-branched DPs.

Linear rheological measurements were performed was performed on a custom-made rheometer, mgRheo[11].

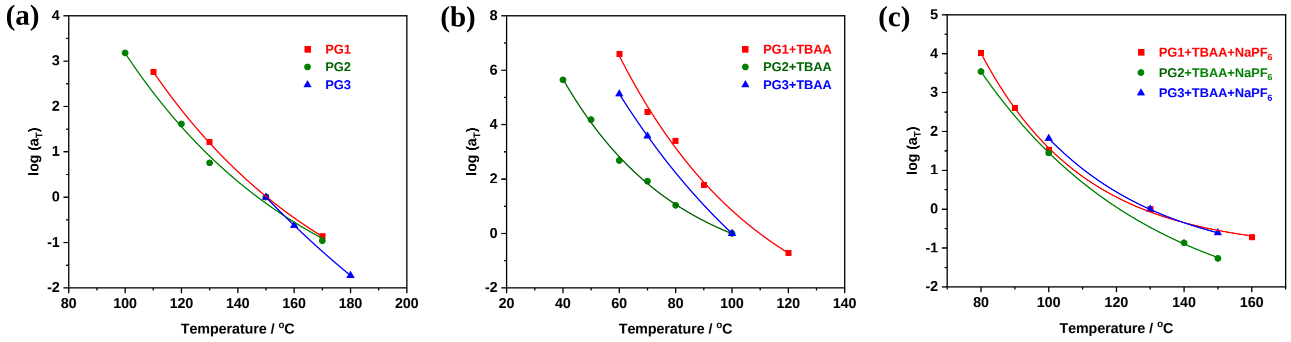

Supplementary Fig. 60 Temperature dependence of horizontal shift factors (a_T) of rotaxane-branched DPs (a) **PGn**, (b) **PGn** + TBAA, and (c) **PGn** + TBAA + $NaPF_6$. The lines are WLF fits to equilibrium data.

To further understand how the changes in the polymer structures influences thermal and rheological properties of the rotaxane-branched DPs. The proposed schematic illustration was shown in Supplementary Fig. 61, after the addition of TBAA, the movement of pillar[5]arene macrocycles in the rotaxane dendrons from urea moiety to the alkyl chain station leads to the enhanced thickness and flexibility of the side rotaxane dendrons. Along with such switching process, local conformations, the volume and rigidity of the polymer chains, and the interactions between the individual DPs has also been regulated, thus further influencing the thermal and rheological properties of the rotaxane-branched DPs.

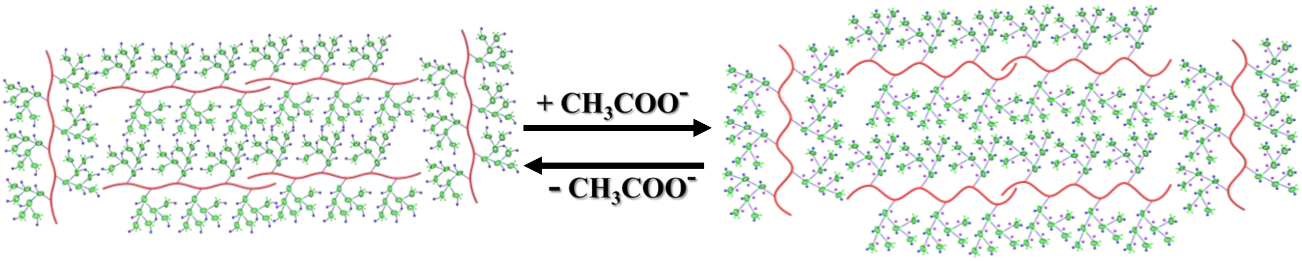

Supplementary Fig. 61 The proposed cartoon representation of tunable thermal and rheological properties of the rotaxane-branched DP **PG3** through the anion-induced switching of the rotaxane branches.

10. Supplementary references

1. Wang, X.-Q. *et al.* Construction of Type III-C Rotaxane-Branched Dendrimers and Their Anion-Induced Dimension Modulation Feature. *J. Am. Chem. Soc.* **141**, 13923-13930 (2019).
2. Love, J. A., Morgan, J. P., Trnka, T. M. & Grubbs, R. H. A practical and highly active ruthenium-based catalyst that effects the cross metathesis of acrylonitrile. *Angew. Chem. Int. Ed.* **41**, 4035-4037 (2002).
3. Johnson, J. A. *et al.* Core-Clickable PEG-*Branch*-Azide Bivalent-Bottle-Brush Polymers by ROMP: Grafting-Through and Clicking-To. *J. Am. Chem. Soc.* **133**, 559-566 (2011).
4. Řezáč , J. & Hobza, P. Advanced Corrections of Hydrogen Bonding and Dispersion for Semiempirical Quantum Mechanical Methods. *J. Chem. Theory Comput.* **8**, 141-151 (2012).
5. Stewart, J. J. P. Stewart Computational Chemistry—MOPAC; Colorado Springs, CO, USA, 2016.
6. Lu, T. & Chen, F., Multiwfn: A multifunctional wavefunction analyzer. *J. Comput. Chem.* **33**, 580-592 (2012).
7. Humphrey, W. Dalke, A. & Schulten, K. VMD: visual molecular dynamics. *J. Mol. Graph.* **14**, 33-38 (1996).
8. Ilavsky, J. & Jemian, P. R. *Irena*: tool suite for modeling and analysis of small-angle scattering. *J. Appl. Crystallogr.* **42**, 347-353 (2009).
9. www.sasview.org
10. Beaucage, G. Approximations Leading to a Unified Exponential/Power-Law Approach to Small-Angle Scattering. *J. Appl. Crystallogr.* **28**, 717-728 (1995).
11. Wu, W. *et al.* Micronewton shear rheometer performing SAOS using 2 mg of sample. *J. Rheol.* **67**, 207-218 (2023).
